# A Biomimetic Twisting Strategy Enables Efficient Electrocatalytic Oxidation of Energy-Dense Hydrazine Hydrate on FeN_2+2_C_4+4_ Sites

**DOI:** 10.1021/jacs.5c08450

**Published:** 2025-08-21

**Authors:** Inbal Offen-Polak, Nagaprasad Reddy Samala, Tomer Y. Burshtein, Syeda M. Zahan, Shuting Xiang, Yair Shahaf, Chen Studnik, Lingmei Ni, Mario U. Delgado-Jaime, Ulrike Kramm, Dario R. Dekel, Charlotte Vogt, Anatoly I. Frenkel, Ilya Grinberg, David Eisenberg

**Affiliations:** † Schulich Faculty of Chemistry, and the Resnick Sustainability Center for Catalysis, 26747 Technion—Israel Institute of Technology, Haifa 3200003, Israel; ‡ The Nancy and Stephen Grand Technion Energy Program, Technion—Israel Institute of Technology, Haifa 3200003, Israel; § Department of Chemistry, 26731 Bar-Ilan University, Ramat Gan 5290002, Israel; ∥ The Wolfson Department of Chemical Engineering, Technion—Israel Institute of Technology, Haifa 3200003, Israel; ⊥ Department of Materials Science and Chemical Engineering, 12301 Stony Brook University, Stony Brook, New York 11794, United States; # Department of Chemistry, 26536 Technical University of Darmstadt, Darmstadt 64287, Germany; ¶ Department of Chemistry, 27802 University of Guadalajara, Guadalajara, Jalisco 44430, Mexico; ∇ Division of Chemistry, Brookhaven National Laboratory, Upton, New York 11973, United States; ■ Israel National Institute of Energy Storage (INIES), Technion—Israel Institute of Technology, Haifa 3200003, Israel

## Abstract

Electrocatalytic hydrazine oxidation holds great promise for enabling fuel cell-powered transportation since hydrazine hydrate (N_2_H_4_·H_2_O) has the highest energy density of all liquid, CO_2_-free fuels (3.45 kWh/L), and the highest fuel cell voltage (1.56 V vs O_2_). Inspired by the ruffling of catalytic centers in oxidative enzymes, we designed a twisted single-atom nanozyme comprising twisted FeN_2+2_C_4+4_ sites, enabling high accessibility of N_2_H_4_ and OH^–^ reactants. Experimental evidence shows that this nanozyme catalyst achieves the lowest oxidation overpotential of all Fe–N–C materials, both in the lab and in direct hydrazine fuel cells, with an open-circuit voltage of 0.95 V, unprecedented for an Fe-based anode. The structure of the catalytic site is elucidated through a combination of electrochemistry, ^57^Fe Mössbauer spectroscopy, high-resolution transmission electron microscopy, and X-ray absorption spectroscopy with crystal field multiplet simulations and fits of the pre-edge features, as well as density functional theory calculations and theoretical simulations of X-ray absorption near edge structure. The experimental and theoretical methods reveal that twisting the active site also shifts its oxidation potential positively and improves N_2_ bubble removal while limiting ammonia production to less than 10 ppm. This work demonstrates the potential of active site twisting to enhance the oxidation of energy-rich and liquid substrates, representing a crucial step toward building a sustainable society.

## Graphical abstract

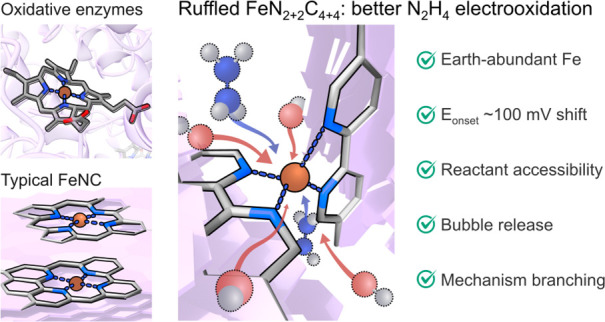

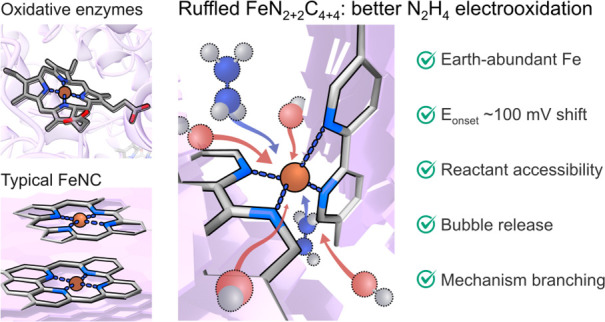

## Introduction

High volumetric energy density is the key fuel requirement for most fuel cell applications, ranging from transportation to stationary power generation.^1–4^ Among liquid fuels, hydrazine (N_2_H_4_) has the highest theoretical cell voltage at 1.56 V against oxygen (compared to 1.23 V for H_2_) and the highest volumetric energy density of all CO_2_-free fuels at 3.45 kWh/L.^5^ These properties make hydrazine particularly attractive for carbon-neutral energy systems, as depicted in Figure 1.^6^ Unlike hydrogen, hydrazine is fully miscible with water, and hydrazine hydrate (N_2_H_4_·H_2_O, 64 wt % hydrazine) can be transported easily and safely.^7^ Despite these advantages, direct hydrazine fuel cells (DHFCs) have faced significant challenges that have hindered their development. While extensively studied in academic, industrial, and military laboratories during the 1970s, the momentum of DHFC research diminished due to the lack of suitable catalysts for its electrooxidation during fuel cell operation.^7–12^ Systems relying on platinum group metals (PGMs) exhibit open-circuit voltages (OCVs) of around 1.0 V, as summarized in Table S1. However, while PGM catalysts are stable and selective for the full 4e^–^ oxidation of hydrazine,^12^ their scarcity makes widespread adoption highly challenging, as has become clear in proton exchange membrane fuel cells.^13^ In later decades, important advances were made with first-row transition metals such as Co and Ni. Although they electrooxidize hydrazine at low overpotentials, Ni and Co are critical raw materials due to geopolitical challenges.^14^ Moreover, they promote the undesired decomposition of N_2_H_4_ to toxic NH_3_ ^15^ and tend to deactivation by surface oxidation, resulting in low electron recovery (∼2 e^–^/N_2_H_4_) and incomplete fuel utilization.^12^

**Figure 1:**
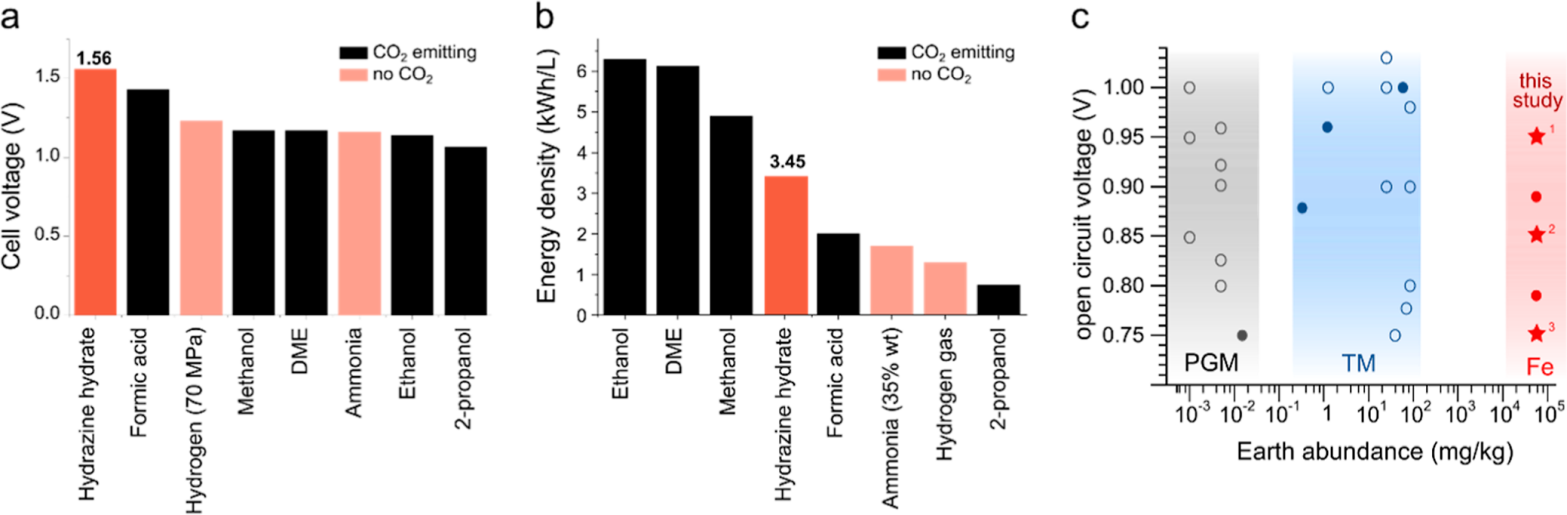
Hydrazine hydrate as an energy-dense liquid fuel: (a) theoretical cell voltages of fuel cells oxidizing different fuels and reducing O_2_. (b) Volumetric energy densities of different liquid fuels (Wh/L). Black bars indicate a CO_2_-producing fuel (see Table S2 for full data). (c) Representative values of OCVs of DHFCs, plotted against the earth crust abundance of the least abundant element in the catalyst, and grouped into PGMs, transition metals (TMs) (other than Fe) and Fe-based catalysts (see Table S1 for full data). Empty circles mark catalysts with elements that catalyze the undesired chemical decomposition of hydrazine to ammonia (Co, Ni, Pt, Ru, and Rh). This study (stars): (1) **FeN**_**4**_ **-twist** anode with the Pt cathode; (2) **FeN**_**4**_ **-twist** anode with the non-PGM CoFe-NC cathode; (3) **FeN**_**4**_ **-flat** anode with the Pt cathode.

In contrast, iron is the fourth most abundant element in the earth crust, at 56 × 10^3^ ppm (Figure 1c), more than other transition metals (10–10^3^ ppm) or PGMs (1–10 ppb). Iron has been largely neglected in the search for DHFC anode catalysts, as manmade carbon-embedded Fe sites have shown only moderate activity toward hydrazine electro-oxidation.^16,17^ However, Fe-based catalytic sites are central to oxidative enzymes in the nitrogen cycle, such as hydrazine dehydrogenase and hydroxylamine oxidoreductase.^18,19^ In these enzymes, the catalytic FeN_4_C_12_ heme centers adopt a twisted (“ruffled”) conformation, where nitrogen-containing rings are twisted in opposite directions along the N–Fe–N axis (see Figure 2a).^20^ While the functional role of heme distortions into ruffled, dome-, or saddle-shaped structures is not fully elucidated,^21^ ruffling is nevertheless highly conserved in oxidative enzymes. This led to suggestions that ruffling affords a stronger (more positive) oxidizing potential through relative stabilization of the ferric (Fe^3+^) state over the ferrous (Fe^2+^) state^22^ and that it modifies the binding of substrates.^23^

**Figure 2:**
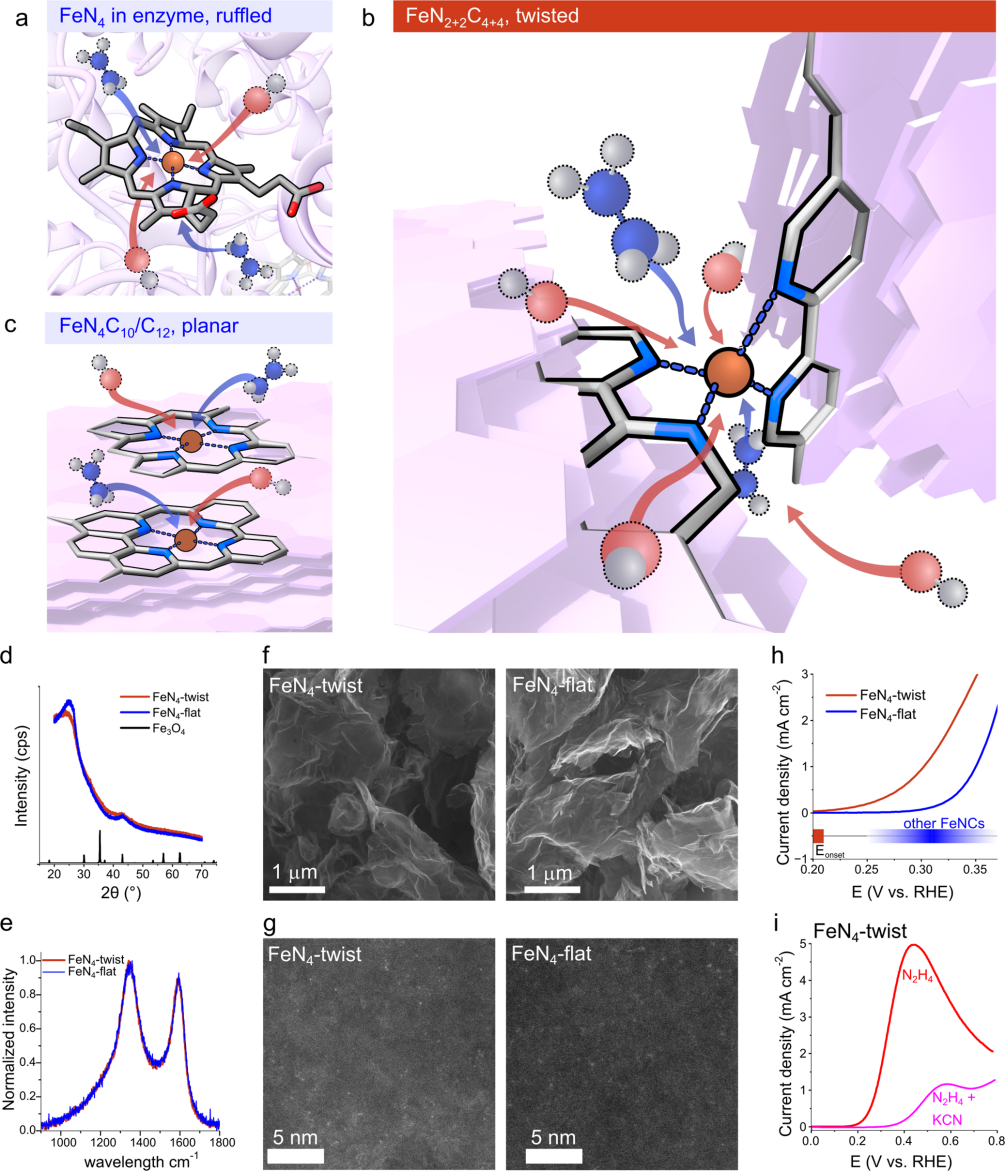
Creating a ruffled FeN_4_ site for hydrazine oxidation. (a) A ruffled heme featuring in the catalytic site of hydrazine dehydrogenase enzyme: an FeN_4_ center with opposite pyrroles twisted along the N–Fe–N axis. Difference in OH^–^ accessibility between a twisted FeN_2+2_ site (b), and a planar, carbon-embedded FeN_4_ site (c). The two materials as characterized by (d) X-ray diffractograms (compared to magnetite, COD 1526955), (e) Raman spectroscopy, (f) scanning electron microscopy, and (g) STEM-HAADF. (h) Hydrazine oxidation voltammograms on **FeN**_**4**_ **-twist** and **FeN**_**4**_ **-flat** in 1 M KOH, 20 mM N_2_H_4_, 10 mV s^–1^; on the bottom, ranges for typical onsets potentials for hydrazine oxidation on FeNCs (range and markings, Table S3) compared to **FeN**_**4**_ **-twist** (red). (i) Cyanide poisoning of **FeN**_**4**_ **-twist** confirms the role of iron in catalysis (see also Figure S4 for poisoning of **FeN**_**4**_ **-flat**).

Inspired by the hypothesis that ruffling of FeN_4_ sites drives the oxidative ability of nitrogen-cycle enzymes, we sought to emulate this structural element in a heterogeneous electrocatalytic nanozyme. To this end, we designed and synthesized a twisted and open FeN_2+2_C_4+4_ catalytic site embedded in a carbon matrix, as illustrated in Figure 2b. Fe- and N-doped carbons (FeNCs) are electrocatalytically active in the nitrogen cycle (reduction of N_2_ and NO_*x*_),^24–26^ the oxygen cycle (reduction of O_2_, evolution of O_2_ and H_2_),^27–31^ and the carbon cycle (reduction of CO_*x*_).^32–34^ Depending on the preparation method, Fe can be coordinated by four pyrrolic or pyridinic N atoms, embedded in a graphene sheet to give planar FeN_4_C_12_ or FeN_4_C_10_ moieties (Figure 2c).^35–37^ By installing twisted FeN_2+2_ sites onto a crumpled support with many micropores, cracks, and edges, we aimed to increase the exposure of the active sites toward reactants, to help the removal of product bubbles, and possibly to offer additional reactivity pathways. Typical atomically dispersed FeN_4_ centers can electro-oxidize hydrazine with onset potentials of 0.36 ± 0.06 V vs RHE, which is earlier more negative than most Fe-based catalysts but still more positive than Pt (−0.05 V vs RHE).^17,38–40^ Our twisted single-atom nanozyme demonstrated the lowest voltametric onset potential for hydrazine oxidation (0.20 V vs RHE at pH 14) compared to all Fe-based catalysts in the literature (0.36 ± 0.06 V vs RHE, Table S3). Moreover, this translated to the highest OCVs of a DHFC with an Fe-based anode, 0.95 V, which is comparable to values obtained with PGM anodes.^7^ We describe the structure of the new site through a combination of electrochemistry, ^57^Fe Mössbauer spectroscopy, high-resolution electron microscopy, density functional theory (DFT) calculations, and X-ray absorption spectroscopy (XAS) with crystal field multiplet simulations and fits of the pre-edge features, as well as theoretical X-ray absorption near edge structure (XANES) simulations, and compare it to the structure of the planar FeN_4_ sites found in conventional FeNC materials. We show how the twisted and open FeN_2+2_C_4+4_ structure enhances the site accessibility to both N_2_H_4_ and OH^–^ reactants, while also shifting the Fe redox potential positive and accelerating the release of bubbles of N_2_ product.

## Results and Discussion

### Design, Characterization, and Electrochemistry of FeNC Catalysts

To promote the formation of twisted and open FeN_2+2_C_4+4_ sites, we aimed to install FeN_4_ moieties predominantly at the edges of adjacent graphene layers, e.g., in micropores, cracks, and other rough features. To this end, we combined the octahedral (that is, nonplanar) iron precursor tris­(1,10-phenanthroline)-iron with a crumpled reduced graphene oxide (rGO) support that can provide folds, kinks, and micropores for Fe anchoring (see Experimental), yielding material **FeN**_**4**_ **-twist**. A benchmark FeN_4_ control sample was prepared from a planar macrocyclic iron phthalocyanine precursor (**FeN**_**4**_ **-flat**) using known methods.^41^ These two materials show similar features in bulk characterization: both have X-ray diffraction patterns with no Fe-based crystals beyond traces of magnetite (2θ = 43°, Figure 2d), and similar carbon features are obtained in Raman spectroscopy (Figure 2e). It is known that after thermal treatment and acid leaching, most FeNC materials typically contain a mixture of sites, such as FeN_4_C_12_, FeN_4_C_10_, nitrogen sites, and carbon sites, as well as iron-based inorganic particles.^42,43^ The carbon scaffolds are crumpled in a similar fashion in **FeN**_**4**_ **-twist** and **FeN**_**4**_ **-flat**, as seen in scanning electron micrographs (Figure 2f), and show identical electrochemical surface areas (132 ± 7 and 139 ± 23 m^2^ g^–1^, respectively). Both materials contain dispersed iron centers (atoms or nanometric clusters), as revealed by high-angle annular dark-field scanning transmission electron microscopy (HAADF-STEM, Figures 2g and S1). Small clusters are known to modulate the catalytic activity,^44^ but increasing their concentration here (by raising the Fe content) did not affect hydrazine oxidation reaction (HzOR) rates, as seen in Figure S2. Hydrazine oxidation activity was tested in a 3-electrode cell with 1 M KOH and 20 mM hydrazine hydrate. The HzOR overpotentials on benchmark **FeN**_**4**_ **-flat** were typical for FeNC materials (*E*_onset_ = 0.30 V vs RHE_pH=14_, compared to 0.25–0.37 V vs RHE_pH=14_, as collected in Table S3). In contrast, **FeN**_**4**_ **-twist** demonstrated outstanding activity, with an *E*_onset_ over 100 mV more negative than that of other FeNCs, as seen in Figure 2h. This shift, suggesting an altogether different active site, was consistent through many repetitions of the synthesis, as seen in Figure S3. Both catalysts respond strongly to cyanide poisoning, confirming the role of iron at the catalytic site, rather than nitrogen dopants or carbon edge defects, as seen in Figures 2i and S4.^45,46^

### Elucidating the Structure of the Twisted FeN_2+2_C_4+4_ Sites

To investigate the chemical environment of the iron site, ^57^Fe Mössbauer spectra were collected at 5.5 (Figure 3a) and 298 K (Figure S5). The spectra of both **FeN**_**4**_ **-flat** and **FeN**_**4**_ **-twist** can be fitted with two doublets at 298 K and an additional one or two sextets at 5.5 K, respectively. The doublets observed at 5.5 K reveal atomically dispersed iron in two configurations,^47^ with D1 typically assigned to low-spin Fe^II^ or high-spin Fe^III^ in planar FeN_4_C_12_ sites^36,42,43,48^ and D2 to intermediate-spin Fe^II^ in FeN_4_C_10_.^42^ As shown in Figure 3b, the ^57^Fe Mössbauer parameters for the D1 site at 5.5 K in **FeN**_**4**_ **-twist** show lower chemical shift (CS) and higher quadruple splitting (QS) than D1 in **FeN**_**4**_ **-flat** (CS = 0.38 vs 0.48 mm s^–1^, QS = 0.98 vs 0.65 mm s^–1^, respectively), indicating differences in the Fe electron density and structural environment. For the D2 sites, the CS is equal for both materials (0.46 mm s^–1^) and the QS is only 0.3 mm s^–1^ higher for **FeN**_**4**_ **-twist**, indicating similar electron density and a slightly different electric field gradient at the Fe nucleus (Table S4 and Figure S5). The QS and CS of D2 in **FeN**_**4**_ **-twist** at 298 K are within the range of those of a similar Fe^II^N_2+2_/C site (parameters in Table S4).^49^ The differences could arise from variations in geometric arrangements, square planar ligands, or a fifth ligand. The large experimental noise precludes unequivocal elucidation of the Fe sites, and a nonplanar FeN_2+2_ site can be neither confirmed nor excluded.^48,50–52^ The sextets at 5.5 K arise from nonleached iron oxide nanoparticles or clusters (see discussion in the Supporting Information).

**Figure 3:**
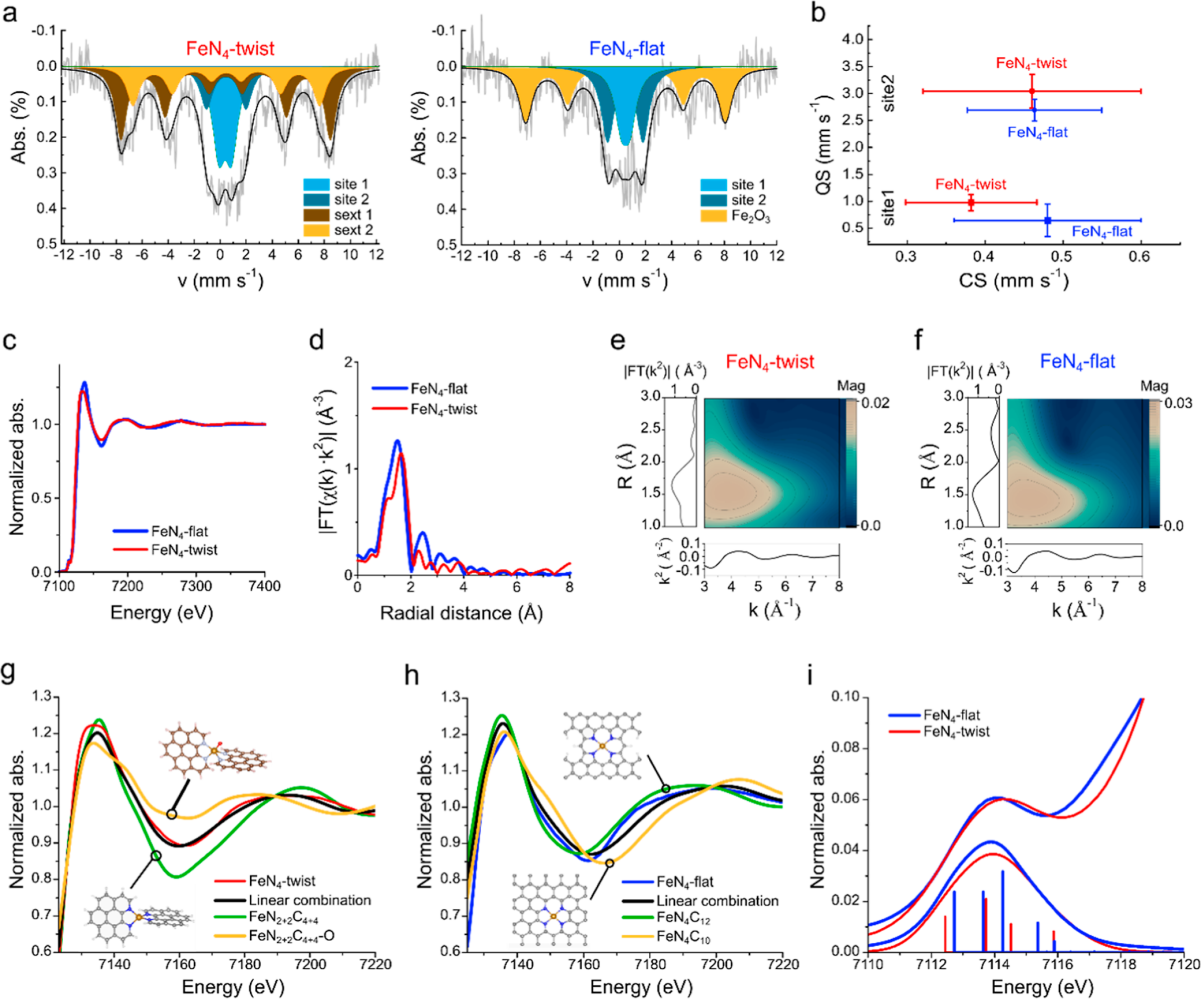
Characterization of the active site in the **FeN**_**4**_ **-twist** and **FeN**_**4**_ **-flat** catalysts. (a) Mössbauer spectra measured at 5.5 K. (b) Mössbauer parameters for both sites at 5.5 K, with typical ranges of QS values.^50^ (c) XAFS spectra and (d) the extended X-ray absorption fine structure (EXAFS) spectra, nonphase corrected (*K*-range 2.8–10). Wavelet transforms of (e) **FeN**_**4**_ **-twist** and (f) **FeN**_**4**_ **-flat**. The fitted linear combination (LCF, red) to the XANES spectra (blue), of the calculated spectra of the suggested structures (green and purple), for (g) **FeN**_**4**_ **-twist** and (h) **FeN**_**4**_ **-flat**. (i) Experimental pre-edge feature of the XANES, compared to representative fit multiplet simulations based on the FeN_4_C_10_ or FeN_4_C_12_ structures for **FeN**_**4**_ **-flat** or the FeN_2+2_C_4+4_ structure for **FeN**_**4**_ **-twist**, noting the additional feature of **FeN**_**4**_ **-flat** between 7116 and 7118 eV arising for *D*_4*h*_ symmetry but missing for **FeN**_**4**_ **-twist** (full assignments are given in Figures S8–S10).

To study the coordination environments of the Fe, both catalysts were characterized by XAS.^53,54^ EXAFS at the Fe edge shows no Fe–Fe bonds at ∼4 Å, corroborating—within the existing limits of detection^55^—the HAADF-STEM observations that Fe is atomically dispersed in both catalysts (Figure 3c,d). This is further confirmed by wavelet transform analysis, where a single feature appears in Figure 3e,f, indicating only Fe–X bonds in both materials and thus a similar atomic composition in the first shell. X-ray photoelectron spectroscopy spectra in the Fe 2p region confirm the presence of Fe–N bonds (Figure S6). Due to the ensemble-average nature of XAS, the spectra of inequivalent sites are weighted in the total spectrum proportionally to their fraction in the sample, making site speciation an ill-posed problem for XAS methods alone.^55^ To prioritize between possible FeN_*x*_ sites predicted by DFT calculations (Table S5), their theoretical XANES spectra were calculated (details are given in the Supporting Information). Subsequently, a search was performed for the combination of spectra that agrees best with the experimental data, in a “forward modeling” approach validated by the success of theoretical spectroscopy codes, such as FDMNES used here,^56^ for reproducing experimental spectra of Fe complexes.^57^ The candidate structures considered included multiple FeN_*x*_ variants from the literature, as summarized in Table S5. For the benchmark **FeN**_**4**_ **-flat** material, the best match with the experimental XANES spectrum is a 1:1 average of the calculated spectra of planar, partially oxygenated FeN_4_C_10_ and FeN_4_C_12_ moieties (Figure 3h), whereas other linear combinations yield significantly poorer fits (as presented in Figure S7).^42^ The formation of such FeN_4_O_*x*_ moieties by pyrolysis of a carbon-supported iron phthalocyanine precursor is expected.^41,51,58^ In contrast, the experimental XANES spectrum of **FeN**_**4**_ **-twist** is best matched by a combination of the FeN_2+2_C_4+4_ moiety, where the nitrogen pairs occupy different planes, and the same moiety but with a coordinated oxygen atom (FeN_2+2_C_4+4_–O), as depicted in Figure 3h. Such FeN_2+2_ sites have been proposed based on evidence from time-of-flight secondary ion mass spectrometry^59^ and further supported by ^57^Fe Mössbauer spectroscopy, XAS, and DFT calculations.^44,50,52,60^

The local environment of FeN_2+2_ sites was further probed by analysis of the pre-edge region of the XANES, since the transition intensity in this region increases in noncentrosymmetric environments (where 4p–3d hybridization is enabled) and can span larger ranges of energy for lower symmetry environments.^61,62^ First, the XANES pre-edge of **FeN**_**4**_ **-twist** and **FeN**_**4**_ **-flat** was analyzed based on the DFT-calculated nonoxygenated structures (FeN_2+2_C_4+4_ for **FeN**_**4**_ **-twist** and FeN_4_C_10_ or FeN_4_C_12_ for **FeN**_**4**_ **-flat**). Crystal field multiplet simulations were fit, based on the methodology of Braun et al.,^63^ suggesting a high-spin Fe­(II) site for **FeN**_**4**_ **-twist** and an intermediate spin Fe­(II) site for **FeN**_**4**_ **-flat** (Figures 3h, S8 and S9). In the case of the DFT-calculated oxygenated structure FeN_2+2_C_4+4_O, which was used to fit the XANES of **FeN**_**4**_ **-twist** in Figure 3g, crystal field multiplet simulations were fit to the pre-edge XANES, confirming a high-spin Fe­(III) site (Figure S10). In the case of **FeN**_**4**_ **-flat**, either of the square planar (*D*_4*h*_) FeN_4_ configuration (C_10_ or C_12_, without O) yielded the observed pre-edge feature, while for **FeN**_**4**_ **-twist**, an out-of-plane (tetrahedral) twist of a high-spin center was found for the nonoxygenated structure. Thus, fitting the multiplet simulations to the pre-edge region verifies the consistency of the models derived from the XANES and Mössbauer analyses.

### Hydrazine Oxidation Mechanism

The enhanced hydrazine oxidation activity of the FeN_2+2_C_4+4_ site can arise from several mechanisms. First, ruffling the FeN_4_ core shifts the redox potential of the Fe positive (more oxidizing), as observed in enzymatic^22^ and molecular iron macrocycles.^64^ Indeed, DFT calculations confirm that the FeN_2+2_C_4+4_ site is easier to oxidize than the FeN_4_C_10_ or FeN_4_C_12_ sites by ∼0.6 eV (Table S6), enhancing the oxidizing ability of the twisted site. Interestingly, recent experiments and calculations have confirmed that the electronic structure of FeN_4_ sites can be affected by adjacent nitrogen defects,^65^ micropores,^66^ or second metals,^67^ leading to enhanced electrocatalysis of oxygen reduction. Twisting can therefore join these methods as a tuning variable for electrocatalytic hydrazine oxidation. In addition to electronic effects, the open FeN_2+2_C_4+4_ site allows OH^–^ to coordinate to iron simultaneously with hydrazine and/or other NH_*x*_ fragments—an impossible feat in planar FeN_4_C_*x*_ moieties. This enables the OH^–^ to deprotonate NH_*x*_ fragments from within both the first and the second coordination spheres, allowing the reaction mechanism to branch in new directions. Indeed, DFT-calculated adsorption energies of intermediates along several HzOR pathways showed that there are more mechanistic options when comparing the FeN_2+2_C_4+4_ site without OH^–^ to the same site but with OH^–^ coordinated (in two different positions, as either an H-bond donor or acceptor to the NH_*x*_ fragment). As shown in Figure 4a (and summarized in Table S7), the reaction pathway without coordinated OH^–^ has the rate-determining step of N_2_* → N_2_(g) (Δ*E* = 0.16 eV), but a switch between pathways—allowing an OH^–^ to coordinate next to the *N_2_ and assist in its desorption—lowers the Δ*E* for this step to −0.08 eV. Such branching of mechanistic pathways is only possible on the open FeN_2+2_ sites, leading to the experimentally observed 100 mV shift in the onset potential on **FeN**_**4**_ **-twist**. Such an effect is indeed more meaningful for the oxidation of bulky N_2_H_4_ than that for the reduction of the smaller O_2_, where the FeN_2+2_ site has lower activity than the FeN_4_ sites.^29,49^

**Figure 4:**
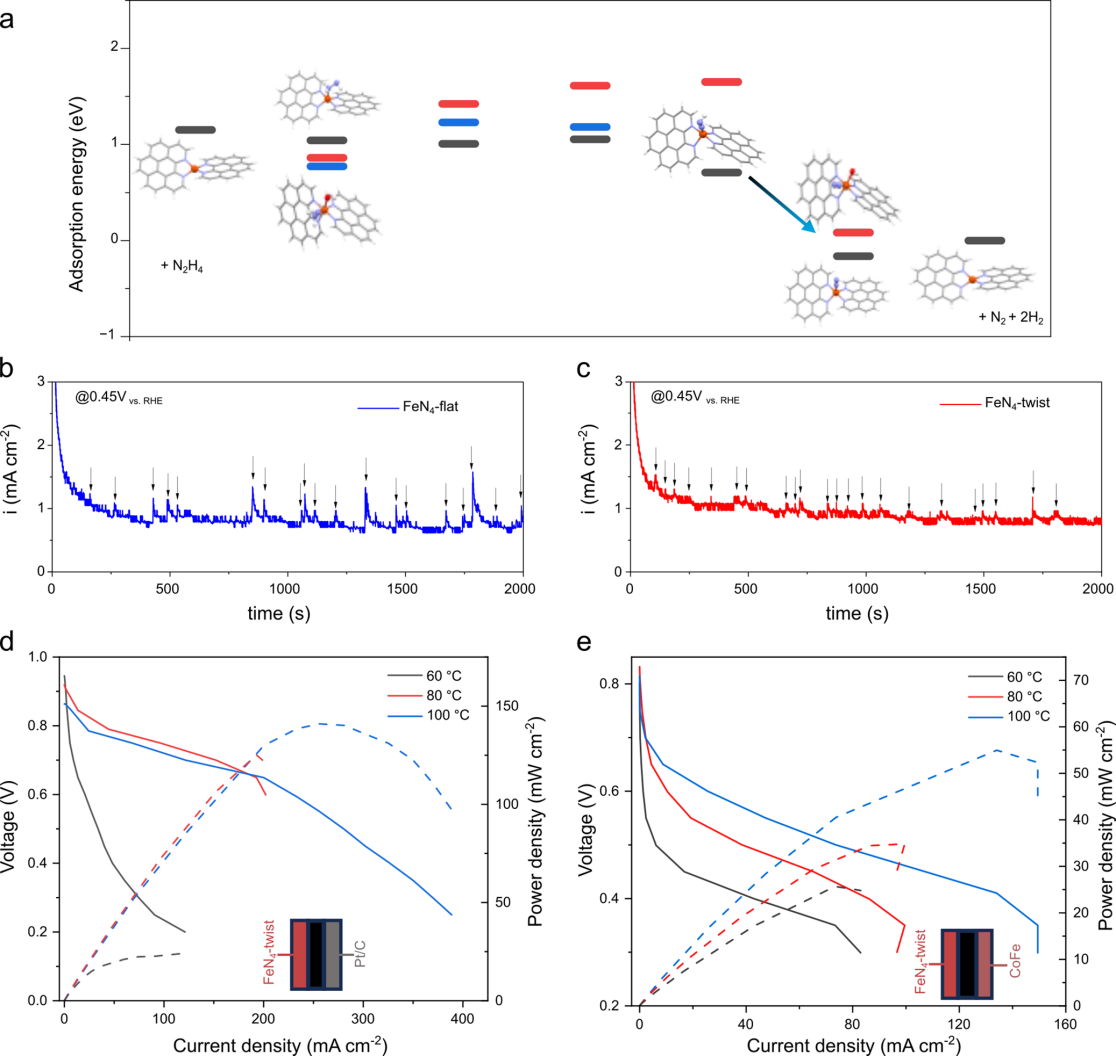
Characterization and fuel cell application of **FeN_4_-twist**. (a) Possible parallel reaction pathways for hydrazine oxidation, showing DFT-calculated adsorption energies of intermediates on FeN_2+2_C_4+4_ (black), and FeN_2+2_C_4+4_–OH in two relative orientations: the OH^–^ is either the H-donor (red) or H-acceptor (blue) to the NH_*x*_ fragment. The arrow indicates the change in the RDS, enabled by the simultaneous coordination of OH^–^ and N_2_ at the open FeN_2+2_C_4+4_ site. (b,c) Chronoamperometric measurements at 0.45 V vs RHE for (b) **FeN**_**4**_ **-flat** and (c) **FeN**_**4**_ **-twist**, marking the current spikes due to N_2_ bubble release. (d) DHFC polarization curves at different temperatures using **FeN**_**4**_ **-twist** anode and Pt cathode with loadings of 4.0 and 0.9 mg cm^–2^, respectively. Inset: a polarization curve in the kinetic region. Cell temperatures of 60, 80, and 100 °C with optimized cathode dew points, in 1 M N_2_H_4_·H_2_O + 4 M KOH, with anode and cathode flow rates of 0.002 and 0.5 L min^–1^ O_2_, respectively. (e) Completely PGM-free DHFC: **FeN**_**4**_ **-twist** anode and CoFe cathode with loadings of 4.0 and 1.0 mg cm^–2^, respectively, in 0.5 M N_2_H_4_·H_2_O + 1 M KOH, with anode and cathode flow rates of 0.002 and 0.5 L min^–1^ O_2_.

The ability of FeN_2+2_C_4+4_ sites to take up edge-position in the carbon will also lead to better accessibility to the reactant stream: a hydrazine molecule approaching a plane-embedded FeN_4_ site can only arrive from one side (defining a hemisphere of approach vectors), while a nonplanar site located in micropores, folds, or edges can be accessed from more directions—up to a full sphere (as visualized in Figure 2b). This idea is further supported by the enhanced current density for the reduction of another molecule, nitrite, on **FeN**_**4**_ **-twist** (Figure S12b), compared to **FeN**_**4**_ **-flat**. Moreover, product removal from different site positions will further differ, enhancing overall reactivity. For example, edge-positioned FeN_2+2_ sites may be more susceptible to flooding by H_2_O, the product of oxygen reduction;^29^ in fact, we did observe lower O_2_ reduction activity on **FeN**_**4**_ **-twist** than that on **FeN**_**4**_ **-flat** (Figure S11a), confirming earlier predictions.^50^ In contrast, the N_2_ gas produced in hydrazine oxidation should be easier to dislodge from edges than from basal planes or water from micropores. To test this idea, we performed a 2 h chronoamperometric experiment at 0.45 V vs RHE, monitoring the current spikes due to the periodic release of N_2_ bubbles (Figure 4b,c). Indeed, the current spikes associated with bubble removal are more regular, more frequent, and smaller in area for **FeN**_**4**_ **-twist** than that for **FeN**_**4**_ **-flat**, indicating improved bubble removal.^68^ Since the macro morphology of the two carbons is identical, as seen in Figure 2e,f, the difference in bubble removal suggests that the N_2_-producing sites in **FeN**_**4**_ **-twist** are more accessible on the microscopic scale (allowing N_2_ molecules to leave more easily) and the mesoscopic scale, e.g., being positioned near edges, cracks, or larger pores, where N_2_(g) bubbles do not get pinned. At even higher potentials >0.45 V vs RHE, as seen in Figure S12, **FeN**_**4**_ **-twist** removes the gas continuously, while **FeN**_**4**_ **-flat** still shows distinct current spikes. Moreover, to confirm the critical role of the crumpled graphene in positioning the FeN_4_ site on the support, we deposited the iron phenanthroline precursor on regular graphite powder (yielding **FeN**_**4**_ **-twist-Gr**), rather than that on rGO as in **FeN**_**4**_ **-twist**. The onset potential of **FeN**_**4**_ **-twist-Gr** shifted 50 mV to positive relative to **FeN**_**4**_ **-twist** (as seen in Figure S13), indicating that the twisted FeN_4_ site attaches more easily in the many folds and pores of crumpled rGO sheets. Furthermore, graphite can accept fewer twisted sites than the crumpled rGO, as evidenced by a 60% decrease in the Fe content relative to that of **FeN**_**4**_ **-twist** (measured by ICP-MS, summarized in Table S8). Finally, loading equal amounts of the two iron precursors on rGO yielded 3 times more Fe on **FeN**_**4**_ **-twist** than on **FeN**_**4**_ **-flat**, further indicating that the twisted site can fit better on a crumpled surface. Overall, the source of enhanced hydrazine oxidation activity of FeN_2+2_C_4+4_ sites likely stems from a mixture of several sources: (1) more positive redox potential, enhancing the oxidizing ability of the Fe; (2) enabling branching of mechanism pathways to include OH^–^ in either the first or second coordination sphere, allowing pathways with lower RDS barriers; and (3) higher microscopic accessibility of the open site for the reactant approach and bubble release.

In oxygen reduction electrocatalysis, Fe-based clusters may contribute by modulating the properties of atomically dispersed FeN_*x*_ moieties.^44^ Indeed, some clusters are present, observed occasionally by HRTEM (Figure S2) and as sextet 2 in the 5.5 K Mössbauer spectrum (Figure 3a). The clusters could arise by deterioration of Fe centers, known to degrade oxygen reduction electrocatalysts.^42,47^ Indeed, the activity of freshly synthesized **FeN**_**4**_ **-twist** degrades somewhat after a few days of air exposure (Figure S14). To gauge the contribution of the clusters to hydrazine oxidation electrocatalysis, we lowered the Fe content in **FeN**_**4**_ **-twist** from 3 to 1 wt % (as determined by ICP-MS and summarized in Table S8). This successfully reduced the cluster content, as evident from typical STEM-HAADF micrographs, while the HzOR activity remained the same at both loadings (Figure S2). This suggests that the role of iron clusters in HzOR electrocatalysis is negligible.

Finally, a DHFC was constructed to test **FeN**_**4**_ **-twist** and **FeN**_**4**_ **-flat** at cell temperatures of 60, 80, and 100 °C with 4 M N_2_H_4_·H_2_O + 1 M KOH solution feed at anode and O_2_ at cathode. High catalytic activity of **FeN**_**4**_ **-twist** was observed in the kinetic region in Figure 4d, with an OCV of 0.945 V at 60 °C (compared to 0.75 V for **FeN**_**4**_ **-flat** in Figure S15). Herein, the advantage in the onset potential of **FeN**_**4**_ **-twist** over **FeN**_**4**_ **-flat** in an RDE experiment was successfully transferred to a real membrane electrode assembly set up of a DHFC, with an ∼200 mV higher OCV. The OCV of 0.945 V is on par with the benchmark PGM catalysts and is the highest observed for an HzOR electrocatalyst based on Fe, the most abundant metal on earth. Unlike with other transition metals, no meaningful amount of ammonia was measured in the anolyte outlet, with a signal lower than the 10 ppm detection limit (Figure S16). Moreover, a relatively high OCV of 0.84 V was registered even when an entirely PGM-free DHFC was used with a CoFe cathode replacing Pt, as seen in Figure 4e. An increase in the cell temperature up to 100 °C significantly enhances the peak power density and limits current density, indicating faster reaction kinetics and better mass transport (Table S9).

## Conclusions

A twisted single-atom nanozyme was designed by biomimetic ruffling of a carbon-embedded FeN_4_ moiety on crumpled rGO. The twisted and open FeN_2+2_C_4+4_ active site, inspired by ruffled heme cores in oxidative enzymes such as hydrazine dehydrogenase, provides enhanced microscopic accessibility for reactants and better bubble removal during the electro-oxidation of energy-dense hydrazine hydrate. This leads to outstanding electrocatalytic activity, with an 100 mV advance in onset potential compared to planar FeN_4_ structures. The enhanced catalytic performance translated well to a DHFC, where the **FeN**_**4**_ **-twist** material achieved an open-circuit voltage of 0.95 V, comparable to that of benchmark PGM catalysts. By optimizing the nanoscale accessibility of the active site, this study positions earth-abundant FeNC materials as strong contenders for driving hydrazine oxidation electrocatalysis, revitalizing interest in hydrazine as the most energy-dense, carbon-free fuel for sustainable energy applications.

## Supplementary Material



## Notes

Experimental methods; synthesis; characterization details; control experiments; further spectroscopic, microscopic, electrochemical, and computational data; analysis; and discussions (PDF)
