# Supplementary material for: A Biomimetic Twisting Strategy Enables Efficient Electrocatalytic Oxidation of Energy-Dense Hydrazine Hydrate on FeN2+2C4+4 Sites
Source: J Am Chem Soc. 2025 Aug 21;147(35):31731–40. doi: 10.1021/jacs.5c08450 (PMC12412151; doi:10.1021/jacs.5c08450)
Supplement: Supplementary file 1 [file ja5c08450_si_001.pdf]

Supporting Information

A biomimetic twisting strategy enables efficient electrocatalytic oxidation of energy-dense hydrazine hydrate on $FeN_{2+2}C_{4+4}$ sites

Inbal Offen-Polak,[a,b] Nagaprasad Reddy Samala,[c] Tomer Y. Burshtein,[a,b] Syeda M. Zahan,[b,d] Shuting Xiang,[e] Yair Shahaf,[a,b] Chen Studnik,[a,b] Lingmei Ni,[f] Mario U. Delgado-Jaime,[g] Ulrike Kramm,[f] Dario R. Dekel,[b,d] Charlotte Vogt,[a,b] Anatoly I. Frenkel,[e,h]* Ilya Grinberg,[c]* David Eisenberg[a,b]*

(a) Schulich Faculty of Chemistry, and the Resnick Sustainability Center for Catalysis, Technion – Israel Institute of Technology, Haifa 3200003, Israel
(b) The Nancy and Stephen Grand Technion Energy Program, Technion – Israel Institute of Technology, Haifa 3200003, Israel
(c) Department of Chemistry, Bar-Ilan University, Ramat Gan 5290002, Israel
(d) The Wolfson Department of Chemical Engineering, Technion – Israel Institute of Technology, Haifa 3200003, Israel
(e) Department of Materials Science and Chemical Engineering, Stony Brook University, Stony Brook, NY 11794, USA
(f) Department of Chemistry, Technical University of Darmstadt, Darmstadt 64287, Germany
(g) Department of Chemistry, University of Guadalajara, Guadalajara Jal. 44430, México
(h) Division of Chemistry, Brookhaven National Laboratory, Upton, NY 11973, USA

Experimental

1. Synthesis

***FeN_4-twist**:* 13.8 mg Fe(II) acetate (0.08 mmol) and 100.3 mg 1,10-phenantroline monohydrate (0.5 mmol) were stirred in ethanol for 20 minutes. Then, the solution was transferred to a vial containing 70 mg of single layer graphene (SLG ,ACS material) and stirred while evaporating the ethanol at 60 °C. The dry slurry was heat-treated in a tube furnace at 600 °C (10 °C/min.) for 2 hours, then naturally cooled to room temperature. The carbon was then acid-leached in 4M HCl overnight, to remove the unstable Fe components. The leached carbon was then filtered and washed with copious amount of doubly distilled water, and then dried at 50 °C. For **FeN_4-twist-1%** the same procedure was used, only the amount of the precursors was adjusted to 2.76 mg Fe(II) acetate and 20 mg 1,10-phenantroline monohydrate. For **FeN_4-twist-Gr** the same procedure was used, but the ratio of the precursors and the carbon substrate was adjusted, to account for the 20 times decrease in surface area. Here, 9.857 mg Fe(II) acetate and 71.64 mg 1,10-phenantroline monohydrate were used for 1.0 g of graphite (Fisher Chemical).

***FeN_4-flat**:* 39 mg Fe(II) phthalocyanine was dissolved in 20 ml DMF. Then, the solution was transferred to a vial containing 60 mg of single layer graphene (ACS material) and stirred while evaporating the DMF at 155 °C. The dry slurry was heat-treated in a tube furnace at 600 °C (10 °C/min.) for 2 hours, then naturally cooled to room temperature. The carbon was then acid-leached in 4M HCl overnight, to remove the unstable Fe components. The leached carbon was then filtered and washed with DI water, then dried at 50 °C. For **FeN_4-flat-Gr** the same procedure was used, but the ratio of the precursor and the carbon substrate was adjusted, to account for the 20 times decrease in surface area. Here, 32.5 mg Fe(II) phthalocyanine were used for 1.0 g of graphite.

2. Electrochemistry

Electrochemical hydrazine oxidation was performed at room temperature in a three-electrode cell connected to a bipotentiostat (BioLogic 600). Catalyst-coated glassy carbon electrode (GCE), a graphite rod and a reversible hydrogen electrode (RHE) were used as working, counter and reference electrodes, respectively. For the catalyst ink, 10 mg of the catalyst sample was dispersed in a mixture of 500 µL ethanol and 10 µL of Nafion solution in IPA (0.5 wt%) and sonicated for 30 minutes. 10 µL of the ink were deposited on GCE (A = 0.1962 cm^2) and dried at 50 °C in air. The mass loading on the working electrode was 0.01 mg. Ar-purged 1M KOH (pH=14) electrolyte solutions were employed for HzOR experiments, where 20mM of hydrazine hydrate (Fisher Scientific) were added. Cyclic voltammetry (CV) and linear sweep voltammetry (LSV) were carried at a potential range from 0 to 0.8V (vs. RHE) with 10 mV s^{-1} scan rate. Prior to the addition of hydrazine, a wetting sequence was performed by cycling the electrodes in the aforementioned potentials, and the background currents were later subtracted from the HzOR CV. Chronoamperometry was used to study bubble release. The N_2(g) produced must originate from hydrazine oxidation, rather than, say the oxidation of NH_3 produced by hydrazine disproportionation: first, because no NH_3 was detected (Figure S16), ruling out disproportionation of N_2H_4 to N_2 and NH_3; second, the catalyst cannot oxidize NH_3 (Figure S11c).

Electrochemical active surface area (ECSA) measurements were performed from the double-layer capacitance of the materials. The capacitance was determined from the CV cycles at 8 scan rates (5,

10, 20, 50, 100, 250 mV s^{-1}) at a small potential window of 0.4 V to 0.7 V (vs. RHE) in 1 M KOH, Ar-purged solutions, where no Faradaic processes are occurring (Figure S19). The following equation was used: $A_{ECSA} = Double\ layer\ capacitance/\ 40\ \mu F\ cm^{-2}$. This calculation assumes a typical value of 40 mF cm^{-2} for the surface-area normalized capacitance associated with double-layer charging.[1] The reported values are the average values measured for at least 4 batches for each catalyst. Poisoning tests of catalysts were recorded for electrodes in either the HzOR electrolyte solution or poison solutions (20 mM of KCN in 1 M KOH+20mM hydrazine hydrate), after an electrochemical wetting process, to ensure the spread of the solutions inside the porous electrode structure.

3. Material Characterizations

Powder X-ray diffraction (XRD) was recorded on Rigaku SmartLab 9 kW X-ray diffractometer utilizing Cu α radiation (1.541 Å). Measurement conditions: PB mode, 45kV, 200mA, step size: 0.01, 2.5 deg/min, 2θ range of 20° to 70°. High-resolution scanning electron microscopy (HRSEM) were performed on a Zeiss-ultra+ (4 kV, in-lens detector) and high-resolution transmission electron microscopy on a FEI Titan Themis (200 kV). Elemental analysis was performed by Mikroanalytisches Labor Kolbe using inductively coupled plasma mass spectroscopy (ICP-MS, Elementar model Vario Mikro Cube) with a CHN analyzer to determine N, while Fe was determined by an ICP-OES (Spectro Model Spectro Arcos). Raman spectroscopy was performed on a Horiba LabRam HR Evolution Raman microscope using ×10 lens, 532 nm laser excitation wavelength, and 1800 grating.

^{57}Fe Mössbauer spectra were recorded in transmission mode using a spectrometer equipped with ^{57}Co/Rh sources. Measurements were carried out at 298 K and 5.5 K in a velocity range of ± 8.8 mm s^{-1} and ± 12.5 mm s^{-1}, respectively. In principle, for FeNC catalysts with such low iron content, it is desired to use as high area loading as possible during spectra acquisition. However, for **FeN_4-twist**, due to its very low density, only 23 mg was possible to be used (Cu holder, 1.0 cm in diameter, 1.0 cm in high) and sealed with Kapton tape for the measurements. For **FeN_4-flat**, 37.7 mg of material was sealed in the same manner as **FeN_4-twist** for the 5.5 K measurement, and to save measurement time for the 298 K spectrum, a larger polytetrafluoroethylene (PTFE) holder (2.5 cm in diameter, 1.2 cm in height) was chosen so that 281 mg of the catalyst material was mounted for the measurement. Spectra were velocity calibrated using the sextet lines of an α-Fe foil and data were fitted using the Recoil software.[2]

X-ray absorption spectroscopy was performed at the SuperXAS beamline (X10DA) at the Swiss Light Source in fluorescence mode. The X-ray beam from the bending magnet was monochromatized with a liquid N_2-cooled Si(111) channel-cut crystal in the QuickXAS monochromator. The Si(111) crystal was rotated at a frequency of 1 Hz across the Fe K-edge, and the signals of the ionization chambers and the angular encoder were sampled at a frequency of 2 MHz. The edge energy was calibrated using a Fe foil. XAS data was evaluated using the ProQEXAFS and Athena packages.

4. Direct hydrazine fuel cell testing

Materials: **FeN_4-twist** and **FeN_4-flat** were used as the anode catalysts. The cathode catalyst Pt/C (60 wt.% on carbon black) was purchased from Alfa Aesar. The cathode catalyst CoFe was synthesized following a published method.[3] PiperION anion exchange ionomer (5 wt% in ethanol) and gas diffusion layer Toray 060 carbon paper with 20% wet proofing were purchased from FuelCellStore (US). Anion exchange membrane low-density polyethylene (LDPE) functionalized with benzyl trimethylammonium

(ion exchange capacity of 2.87 ± 0.05 mmol g^{-1}, hydroxide conductivity of 145 mS/cm at 80 °C, and hydrated membrane thickness of 50 µm) was used as membranes in the DHFC tests.[4,5] Oxygen with 99.999 % purity was purchased from Maxima, Israel. Hydrazine hydrate (99%) was purchased from Fisher Scientific.

Catalyst ink and gas diffusion electrode (GDE) preparation: GDE preparation has been reported in detail elsewhere.[6–9] In brief, 53 mg of the anode catalyst powder was ground in a mortar for 3 minutes to reduce the agglomeration of the particles. 1.86 mL of deionized water was added to form a slurry, followed by 5 minutes of grinding. 5 mL of isopropanol (IPA) was added to this slurry, and the mixture was ground for an additional 5 minutes. An additional 17.5 mL of IPA was added to the slurry in a volume ratio of water:IPA of 1:12. The mixture was transferred to a centrifuge tube and sonicated for 10 min in an ELmasonic P 60H ultrasonic bath under 180 W, 37 kHz, at ~5 °C. After 10 min, 320 µL of ionomer dispersion was added to reach an ionomer:catalyst weight ratio of 1:4. The resulting catalyst ink was further sonicated in the ultrasonic bath for 2 h. Similarly, for the cathode, 90 mg Pt/C (60 wt%) and 271 µL of ionomer were used. Water and IPA were added in a similar ratio as in the case of the anode catalyst, and the ink was prepared in the same way. The catalyst inks were sprayed onto 2.5 cm^2 sized gas diffusion layers (GDLs) secured onto the heating plate at 90 °C using an ultrasonic Y&D 7300N spray robot. After each sprayed layer, the GDLs were subsequently placed on a heater at 120 °C to dry for 30 sec. The ink was sprayed with an ink flow rate of 0.18 mL/minute and an inert gas pressure of 0.6 bar. The loading of the anode GDEs was 4.0±0.1 mg_{FeNC}/cm^2. The loading of the cathode GDE was 1.0±0.1 mg_{Pt}/cm^2. The thickness of the FeNC anode catalyst layers was similar for all the cells, in the range of 428–535 µm. The thickness of the cathode catalyst layer was *ca.* 25 µm.

Fuel cell tests: The GDEs were immersed in a 2 M aqueous solution of KOH for 12 hours, with an additional 1 h of soaking where the solution was changed every 20 minutes. The anion exchange membrane was soaked in a 1 M aqueous KOH solution for 1 h, with the solution being changed every 20 min to convert it to its hydroxide form. The membrane electrode assembly was then assembled between two 5 cm^2 single-serpentine graphite bipolar flow fields. PTFE gaskets with a cut-out area of 2.5 cm^2 were used to obtain a desired pinch of 30%, and the assembly was pressed using a torque of 4.5 Nm. The fuel cell test was performed using the Greenlight fuel cell test station following the procedures detailed elsewhere.[10–12] For preparing the anode fuel, 224.4 g of KOH pellets and 51.6 ml of 99% stock solution of hydrazine hydrate ($N_2H_4 \cdot H_2O$) were added to 1 L of milli-Q water to make a 1 M $N_2H_4 \cdot H_2O$ + 4 M KOH solution. The hydrazine alkaline solution at 60 °C was supplied to the anode at a flow rate of 0.002 L/min, and humidified oxygen was supplied to the cathode at a flow rate of 0.5 L/min. The cell was first heated up to 60 °C. After the OCV stabilized, a break-in procedure[13,14] was performed by stepping down the voltage at steps of 0.1 V and holding at each voltage until the current stabilized. After the break-in, polarization curves were recorded at a scan rate of 10 mV/s. Polarization curves were further obtained at 80 and 100 °C.

5. Computational studies

Spin-polarized density functional theory (DFT) the calculations were performed using the Quantum Espresso-v6.2 (QE) package with the PBE exchange-correlation functional and the plane wave basis and GBRV pseudopotentials. All calculations were performed at the gamma k-point and ionic relaxations were carried out until the energy variation between the self-consistent field iterations was

less than 10^{-6} Ry and the forces on all atoms were lower than 10^{-6} Ry/au. In all calculations, the kinetic energy cutoff for the plane-wave basis set wavefunction and the charge density cutoff were set to 50 and 300 Ry, respectively. The default Gaussian smearing method was employed with a smearing parameter of 0.01 Ry. Although energy barriers can be calculated by DFT, it is unreliable to correlate them to shifts in E_{onset} when the differences (100 mV) fall within the range of errors that can arise from DFT functional and basis set choice (<0.2 eV or so). Thus, we avoided such direct comparisons between experiment and calculation, instead discussing the trend within each method.

Data Analysis

1. ^{57}Fe Mössbauer spectroscopy

At 298 K, iron oxide particles or clusters give rise to doublets in the ^{57}Fe Mössbauer spectra, because of spin relaxation. At 5.5 K these sites become magnetically ordered, giving rise to sextets (64% in **FeN_4-twist** and 46% in **FeN_4-flat**, Table S4). Such moieties are also suggested by STEM-HAADF and XRD results. Similar sextets have been previously assigned for Fe(III) oxide resulting from oxidation of an FeN_4C_{12} site.[15]

The average spin state *S* can be calculated from relative areas in the 5.5 K ^{57}Fe Mössbauer spectra, under different assumptions. For **FeN_4-twist**, assuming D1 is HS Fe(III), $S = 0.235{\cdot}2.5 + 0.125{\cdot}1 + 0.64{\cdot}2.5 = \underline{2.3}$; or assuming D1 is LS Fe(II), $S = 0.235{\cdot}0 + 0.125{\cdot}1 + 0.64{\cdot}2.5 = \underline{1.7}$. For **$FeN_4$-flat**, assuming D1 is HS Fe(III), $S = 0.22{\cdot}2.5 + 0.32{\cdot}1 + 0.46{\cdot}2.5 = \underline{2.0}$; or assuming D1 is LS Fe(II), $S = 0.22{\cdot}0 + 0.32{\cdot}1 + 0.46{\cdot}2.5 = \underline{1.47}$. This spans a range which accommodates the sites derived from XAS analysis.

2. X-ray adsorption spectroscopy

Linear combination fitting of XANES: XANES calculation was conducted on the DFT-calculated models via FDMNES. Since the XANES of experimental **FeN_4-twist** and the theoretical XANES spectra of $FeN_{2+2}C_{4+4}$ and $FeN_{2+2}C_{4+4}O$ are crossing the isosbestic points as shown in Figure 3g and the experimental XANES of **FeN_4-twist** is in between the theoretical XANES spectra of $FeN_{2+2}C_{4+4}$ and $FeN_{2+2}C_{4+4}O$, it is assumed that sample **FeN_4-twist** contains these two species of $FeN_{2+2}C_{4+4}$ and $FeN_{2+2}C_{4+4}O$. Therefore, linear combination fitting (LCF) was conducted on the sample **FeN_4-twist** with $FeN_{2+2}C_{4+4}$ and $FeN_{2+2}C_{4+4}O$ as two standards. Based on LCF, the **FeN_4-twist** consists of 48.6% $FeN_{2+2}C_{4+4}$ and 51.4% $FeN_{2+2}C_{4+4}O$. Similarly, LCF was conducted on **FeN_4-flat** with FeN_4C_{12} and FeN_4C_{10} as two standards. Based on the LCF, **FeN_4-flat** consists of 51.7% FeN_4C_{12} and 48.3% FeN_4C_{10}. From LCF results we know that both **FeN_4-twist** and **FeN_4-flat** samples are not pure and have two species.

Multiplet ligand-field analysis of pre-edge XAS: We fit crystal field multiplet simulations based on the four structures that are in best agreement with the XANES experiments, using the methodology used recently by Braun *et al.*[16] In this study, the crystal field is derived by approximating the structure of an Fe site to a trigonal symmetry (D_{3h}/C_{3v}), where a trigonal crystal field potential is used. Then, the individual 1*s*-3*d* transitions were computed by operators for quadrupole transitions to specific $d_{x^2-y^2}$, d_{z^2}, d_{xy}, d_{xz} and d_{yz} orbitals, calculated using an expansion on A_{km} coefficients consistent to a cubic symmetry[17,18] (similar to the expansion in spherical harmonics for the 3*d* orbitals in this symmetry). Under this framework, each 1*s*-3*d* transition computed in the multiplet simulation can be projected into transitions to specific cubic 3*d* orbitals, even if the symmetry of the Fe site is different from cubic

or tetragonal (transitions in would reflect a mixing of 3*d* orbitals, even in tetragonal symmetries because of atomic interactions). We adapted this methodology to fit crystal field multiplet simulations to the experimental pre-edge XANES of **FeN_4-flat** based on either FeN_4C_{10} or FeN_4C_{12} structures (both consistent with a local D_{4h} symmetry around Fe); and to fit crystal field multiplet simulations to the experimental pre-edge XANES of **FeN_4-twist** based on the $FeN_{2+2}C_{4+4}$ structure (consistent with a local T_d symmetry), and the $FeN_{2+2}C_{4+4}O$ structure (consistent with an approximately C_{3v} symmetry). Within this approach, the 4*p*-3*d* hybridization contribution was modeled by fitting a specific parameter for each irreducible representation responsible for the mixing. For example, in the case of C_{3v} symmetry, a scaling factor parameter is used to model the mixing of electric dipole characters through $4p_z$-$3d_{z^2}$ mixing, which transforms as a_1 and is enabled by sigma bonding with the ligands.[18,19] In addition, another scaling factor to model the mixing of dipole character through $4p_x$-$3d_{xz}$ and $4p_y$-$3d_{yz}$ hybridizations, which transform collectively as *e* and are enabled by π bonding with the ligands. Thus, two scaling factors were used to model the electric dipole character that is mixed into the 1*s*-3*d* transitions to the *a1 and e* orbitals, respectively, in the case of crystal field simulations based on the $FeN_{2+2}C_{4+4}O$ structure. Furthermore, for a tetrahedral Fe site, the $4p_x$-$3d_{yz}$, $4p_y$-$3d_{xz}$ and $4p_z$-$3d_{xy}$ hybridizations transform as t_2 and are enabled by σ-bonding with the ligands. Thus, a single scaling factor was used to model the electric dipole character that is mixed into the 1*s*-3*d* transitions to the t_2 orbitals in the case of crystal field simulations based on the $FeN_{2+2}C_{4+4}$ structure. Finally, we note that in a square planar geometry (as in **FeN_4-flat**), the multiplet structure spans a larger energy region, while in **FeN_4-twist**, the fit multiplet simulations that are based on the non-oxygenated (Figure S6) or the oxygenated structures (Figure S7) are more contracted.

Supporting tables

Table S1: Open circuit potentials in direct hydrazine fuel cells (operating with O_2) with different anode electrocatalysts. The rarest element component was used for classification in Figure 1c.

**Electrocatalyst**	**OCV (V)**	**Reference**
FeNC	0.89	[20]
FeN-CNT	0.79	[21]
NiFeP/NF	0.98	[22]
Ni	0.8	[23]
Ni/C	0.9	[24]
Ni/C	0.99	[25]
$Ni_{0.87}Zn_{0.13}$	0.78	[26]
NiZn/KB	0.89	[27]
Cu-Ni	0.97	[28]
Cu	0.92	[29]
Cu	1	[30]
$Ni_{0.9}La_{0.1}$	0.75	[31]
Fe-CoS_2	1.03	[32]
Cu@NiCo/C	0.9	[33]
Ni_3N-Co3N/NF	1	[34]
CoP-NWA@CP	0.91	[35]
Co_6W_6C/C	1	[36]
MoC_x-NC	0.96	[37]
PdCu/C	0.75	[38]
Pt/C on Ni foam anode	0.92	[39]
RuP_2/NC	1	[40]
Se@C-1000	0.88	[41]
Rh-Rh_2O_3/C	0.99	[42]
Pt black	1.2	[43]
Pd	1.1	
Ru	0.9	
Rh	0.85	
Pt/C, Pt/Cu	0.8	[44]
PtAgBiTe/C	0.9	[45]
Pt	0.83	[23]
NiPt/C	0.82	[46]
$CoPt_3$/CoPt⊂PLNC	0.96	[47]

Table S2: Thermodynamic data of fuels for O_2 fuel cells: ΔH enthalpy, ΔG Gibbs free energy, ε theoretical energy conversion efficiency, E^0 theoretical cell voltage and ED energy density (reproduced with corrections from Sakamoto *et al.*[46] and Soloveichik[48]).

**Fuel**	**Conditions**[a]	**Anode products**	**number of e^-**	**-ΔH, kJ mol^{-1}**	**-ΔG, kJ mol^{-1}**	**ε, %**	**E^0, V**	**ED kWh L^{-1}**
H_2	700 atm, 25 °C[b]	H_2O	2	285.8	237.1	83	1.23	1.30
liquid H_2	1 atm, -252.9 °C[c]	H_2O	2	285.8	237.1	83	1.23	2.33
Methanol	1 atm, 25 °C	CO_2 + H_2O	6	726	702	97	1.21	4.82
Ethanol	1 atm, 25 °C	CO_2 + H_2O	12	1367	1325	97	1.15	6.30
2-Propanol	1 atm, 25 °C	$(CH_3)_2CO$[g]	2	216.1	206.5	96	1.07	0.75
Formic acid	1 atm, 25 °C, 88 wt.%[d]	CO_2 + H_2O	2	254.3	270	106	1.40	1.99
Dimethyl ether (DME)	5 atm, 25 °C[e]	CO_2 + H_2O	12	1460.3	1387.2	95	1.20	6.12
Ammonia	1 atm, 15.6 °C, 35 wt%[d]	N_2 + H_2O	3	383	339	89	1.17	1.71
Hydrazine hydrate	1 atm, 25 °C[f]	N_2 + H_2O	4	606	602	99	1.56	3.45 [h]

[a] The energy density depends greatly on the physical properties of the fuel i.e. the phase (gas, liquid or in solution?), the pressure, temperature and concentration. The values estimated here are the highest theoretical values, but they do consider practical applications. For example, ammonia and hydrazine fuel cells would use aqueous solutions for the fuel, for safety concerns.

[b] The pressure used to pressurize hydrogen for fuel cells.

[c] At the boiling point.

[d] Common concentrations for highly concentrated solution that could be used for a liquid fuel cell.

[e] The pressure required for liquification.

[f] The hydrate form is used as it is safer and more practical to use for fuel cells.

[g] 2-propanol oxidation is highly selective towards acetone as the final product.[49–53]

[h] This number has been calculated using the molar mass and density values for hydrazine hydrate ($N_2H_4 \cdot H_2O$) 50.061 g/mol and 1.032 g/ml, respectively.

Table S3. Voltametric onset potentials for hydrazine oxidation on FeNC electrocatalysts in the literature

**Catalyst**	**Onset potential, V *vs.* RHE**	**Reference**
Fe_3C-embedded N-doped carbon (wash-NCFe)	0.35	[54]
Fe_3C/NC	0.38	[55]
FeN_x SAC on CNTs (FeSA/CNT)	0.32	[21]
FeN_x SAC on carbon (FeN_4/HPCM)	0.25	[20]
FeN_x SAC on carbon (Fe-NC-2-1000)	0.25	[56]
wash-Fe_2MoC/NC	0.28	[57]
FeN_x SAC on carbon, FeNC-Eu	0.32	[58]
FeNC-Tb	0.34	
FeNC-Yb	0.38	
FeNC-Sm	0.38	
FeNC-Nb	0.38	
FeNC-Er	0.38	
FeNC-La	0.38	
Fe_2O_3/C	0.50	[59]
**Average ± standard deviation**	**0.36 ± 0.06**	
FeN_4-flat	0.30	This work
**FeN_4-twist**	**0.20**	**This work**

Table S4: Summary of Mössbauer parameters measured at 5.5 K and at 298 K, and their suggested assignments.

		CS (mm/s)	ΔE_Q (mm/s)	H (T)	FWHM	Area (%)	Possible assignment	Ref.
**FeN_4-twist**	doubl1	0.38 ± 0.08	0.98 ± 0.15		1.2*	23.5± 3.1	Fe(III)-N_4 high spin or Fe(II)-N_4 low spin	[60]
		0.36 ± 0.03	0.85 ± 0.07		0.84	90 ± 22		
	doubl2	0.46 ± 0.14	3.04 ± 0.31		1.2*	12.5 ± 3.1	Fe(II)-N_4 intermediate spin	[60]
		0.42*	2.5 ± 1.3		0.96	10 ± 24		
	sext1	0.42 ± 0.06		49.92 ± 0.67	1.14	41 ± 17	α-Fe_2O_3 (or γ-Fe_2O_3)	[61]
	sext2	0.47 ± 0.10		44.50 ± 1.50	1.18	23 ± 16	Iron oxidic species	
**FeN_4-flat**	site1	0.48 ± 0.12	0.65 ± 0.30		1.2*	22.1 ± 5.0	Fe(III)-N_4 high spin or Fe(II)-N_4 low spin	[60]
		0.37 ± 0.03	0.66 ± 0.05		0.56	84.8 ± 8.4		
	site2	0.46 ± 0.09	2.69 ± 0.20		1.2*	31.5 ± 4.7	Fe(II)-N_4 intermediate spin	[60]
		0.26	2.65 ± 0.37		0.56*	15.2 ± 7.5		
	sext2	0.47 ± 0.95		47.14 ± 0.55	1.2*	46.3 ± 7.1	Iron oxidic species	[61]

Table S5: The structures considered for XANES spectra fitting.

	**FeNC structure**	**FeNC oxygenated structure**
**$FeN_{2+2}C_{4+4}$**	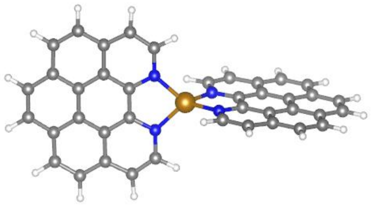	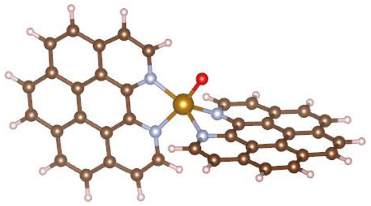
**FeN_4C_{12}**	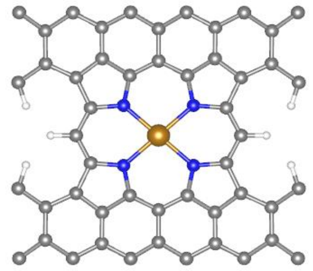	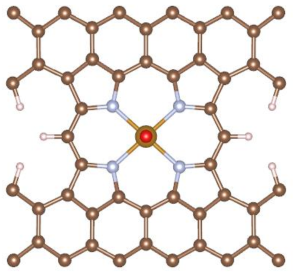
**FeN_4C_{10}**	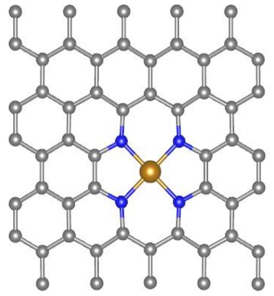	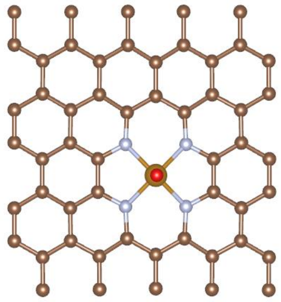
**FeN_2**	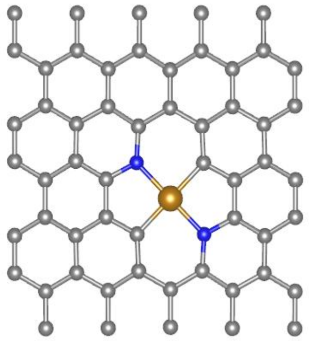	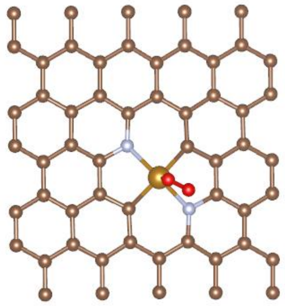
**FeN_4C_8**	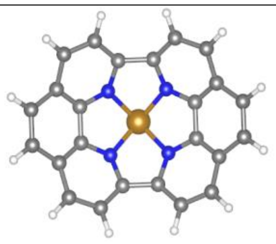	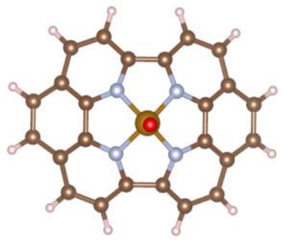
**$FeN_{2+2}C_{4+4}$-planar**	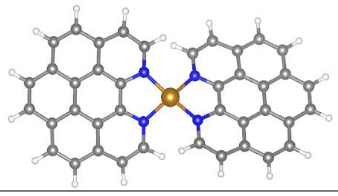	

Table S6: Energy required for 1e⁻ oxidation of each Fe-N_4 site, calculated by DFT at the PBE/6-311++G** level of theory.

	**Energy difference (eV)**
**$FeN_{2+2}C_{4+4}$**	4.85
**FeN_4C_{10}**	5.47
**FeN_4C_{12}**	5.41

Table S7: DFT-calculated adsorption energies of intermediates on $FeN_{2+2}C_{4+4}$ (black), and $FeN_{2+2}C_{4+4}$-OH in two relative orientations: the OH is either the H-donor or H-acceptor to the NH_x fragment. The related structures are presented for each step.

**Adsorbed intermediate**	**Adsorption energy on $FeN_{2+2}C_{4+4}$ (eV)**	**Adsorption energy on $FeN_{2+2}C_{4+4}OH$, when OH is H-acceptor (eV)**	**Adsorption energy on $FeN_{2+2}C_{4+4}OH$, when OH is H-donor (eV)**
**N_2H_4 (gas)**	1.15	1.15	1.15
	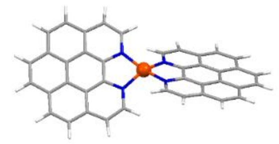	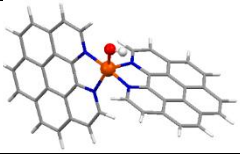	
**N_2H_4**	1.04	0.77	0.86
	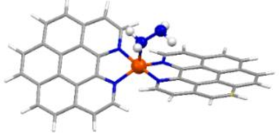	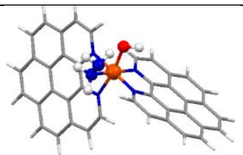	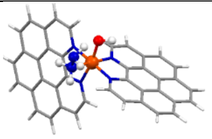
**N_2H_3**	1.01	1.23	1.42
	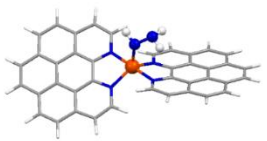	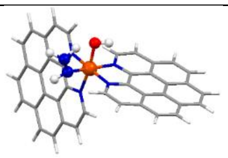	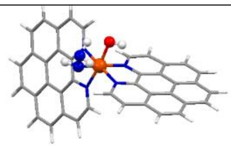
**N_2H_2**	1.05	1.18	1.61
	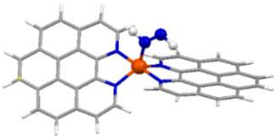	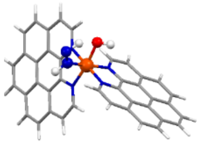	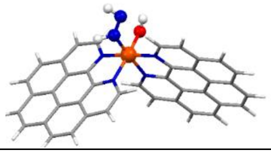
**N_2H**	0.71	1.65	1.65
	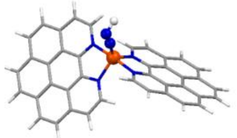	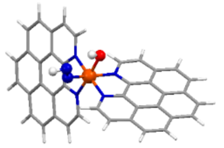	
**N_2**	–0.16	0.08	0.08
	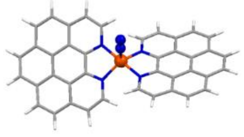	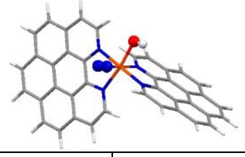	
**N_2 (gas)**	0	0	0
	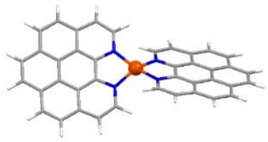	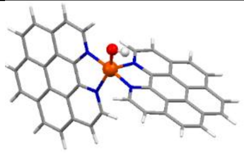	

Table S8. Elemental composition from ICP-MS.

	N wt%	Fe wt%	N/Fe atomic ratio
**FeN_4-twist**	4.56	2.87	6.3
**FeN_4-flat**	3.77	0.95	15.8
**FeN_4-twist-Gr**	0.3	1.12	1.1
**FeN_4-flat-Gr**	0.48	0.38	5.0
**FeN_4-twist-1.2%**	1.61	0.97	6.6

Table S9: Performance of direct hydrazine fuel cells.

**Anode (mg/cm^2)**	**Cathode (mg/cm^2)**	**Cell temperature (°C)**	***j* @ 0.8 V (mA/cm^2)**	***j* @ 0.6 V (mA/cm^2)**	**PPD[b] (mW/cm^2)**	**LCD (mA/cm^2)**	**NH_3 in exhaust (ppm)[a]**
**FeN_4-twist** 4 ± 0.1	Pt/C, 0.9	60	4	30	23	115	<10 ppm
		80	45	97	120	193	
		100	17.5	236	140	388	
**FeN_4-flat** 4 ± 0.1	Pt/C, 0.9	60	0	12.5	38	141	--
		80	0	14	78	197	
		100	0	24	88	331	

[a] Measured by NMR, with a calibration curve (Figure S16).
[b] Peak power density
[c] Limiting current density

Supporting figures

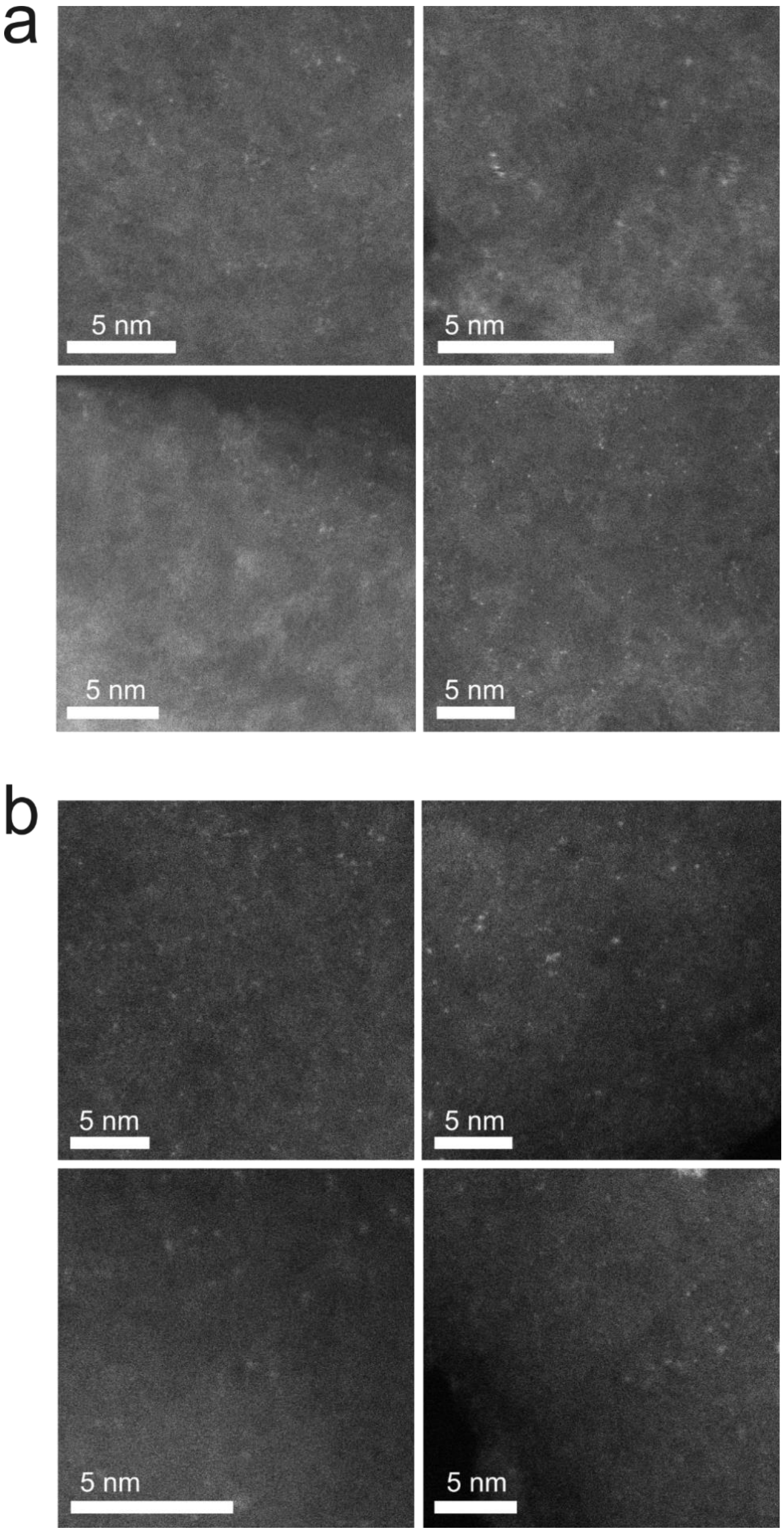

Figure S1: Additional high-angle annular dark-field scanning transmission electron microscopy (HAADF-STEM) micrographs of (a) **FeN_4-twist** and (b) **FeN_4-flat**.

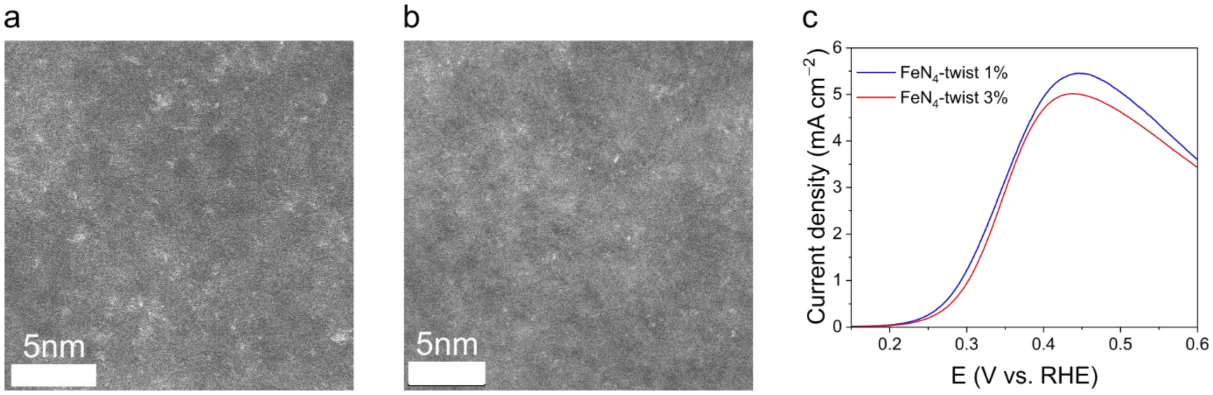

Figure S2: Increasing the Fe loading from 1% in **FeN_4-twist** to 3% in **FeN_4-twist-3%**, and its effect on cluster loading and electrochemistry: Representative STEM-HAADF micrographs demonstrating the dispersion of Fe in (a) **FeN_4-twist-3%**, showing a larger density of clusters, compared to (b) **FeN_4-twist** (1% loading), where Fe is more atomically dispersed. (c) LSV of HzOR electrocatalysis of these **FeN_4-twist** variants prepared with different Fe content (1% and 3% wt, measured by ICP-MS), recorded in 1 M KOH and 20 mM hydrazine.

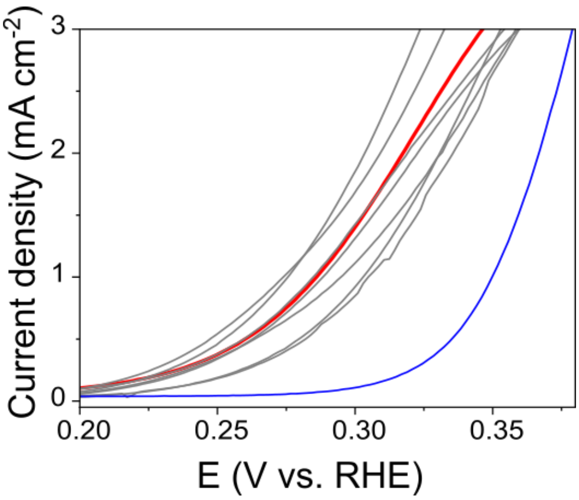

Figure S3: Hydrazine oxidation voltammograms on **FeN_4-twist**, showing that the LSV presented in Figure 3g (red here) is typical by comparing it to LSVs from three different synthesis batches and from several ink preparations of the same material (grey lines). The LSV of **FeN_4-flat** is given for comparison in blue. Measured in 1 M KOH, 20 mM N_2H_4, 10 mV s^{-1}. The excellent batch-to-batch reproducibility of **FeN_4-twist** allowed us to make ~150 mg batches up to 2 gr total.

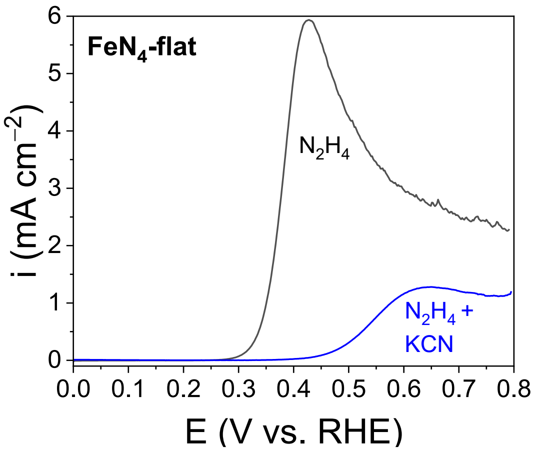

Figure S4: Poisoning tests of **FeN_4-flat**: LSVs were recorded of electrodes in either the HzOR electrolyte solution or poison solutions (20mM of KCN in 1M KOH+20mM hydrazine hydrate), after an electrochemical wetting process, to ensure the spread of the solutions inside the porous electrode structure.

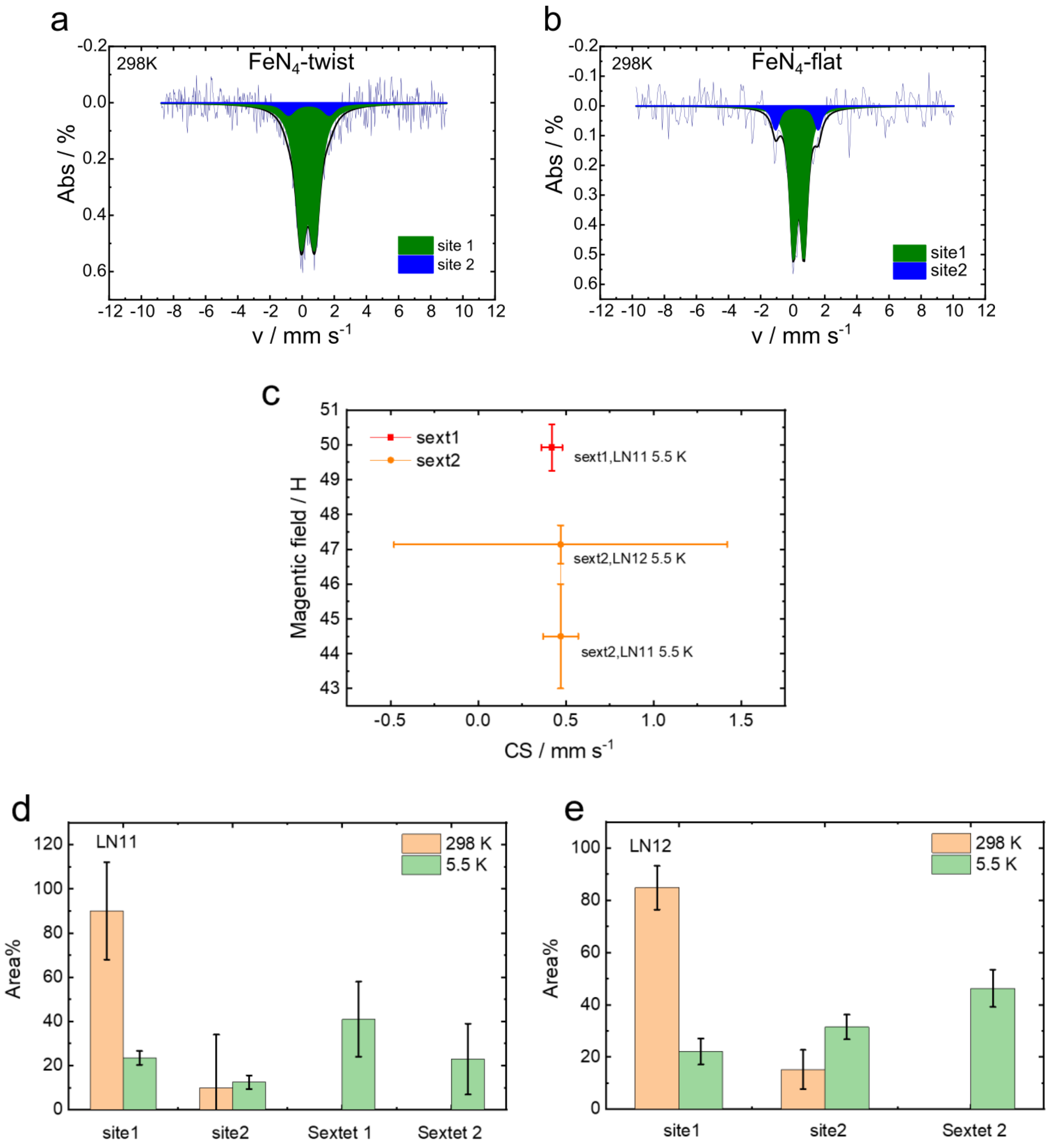

Figure S5: (a,b) ^{57}Fe Mössbauer spectra at 298 K, for **FeN₄-twist** and **FeN₄-flat**, respectively. (c) Analysis of the Mössbauer spectra, in terms of the magnetic field and the chemical shift of the sextets appearing in the spectra of **FeN_4-flat** and **FeN_4-twist** at 5.5 K. (d,e) The percentage of each site, derived from spectra recorded at either 298 K or 5.5 K for each catalyst. By decreasing the temperature, Fe oxides can be distinguished from site 1.

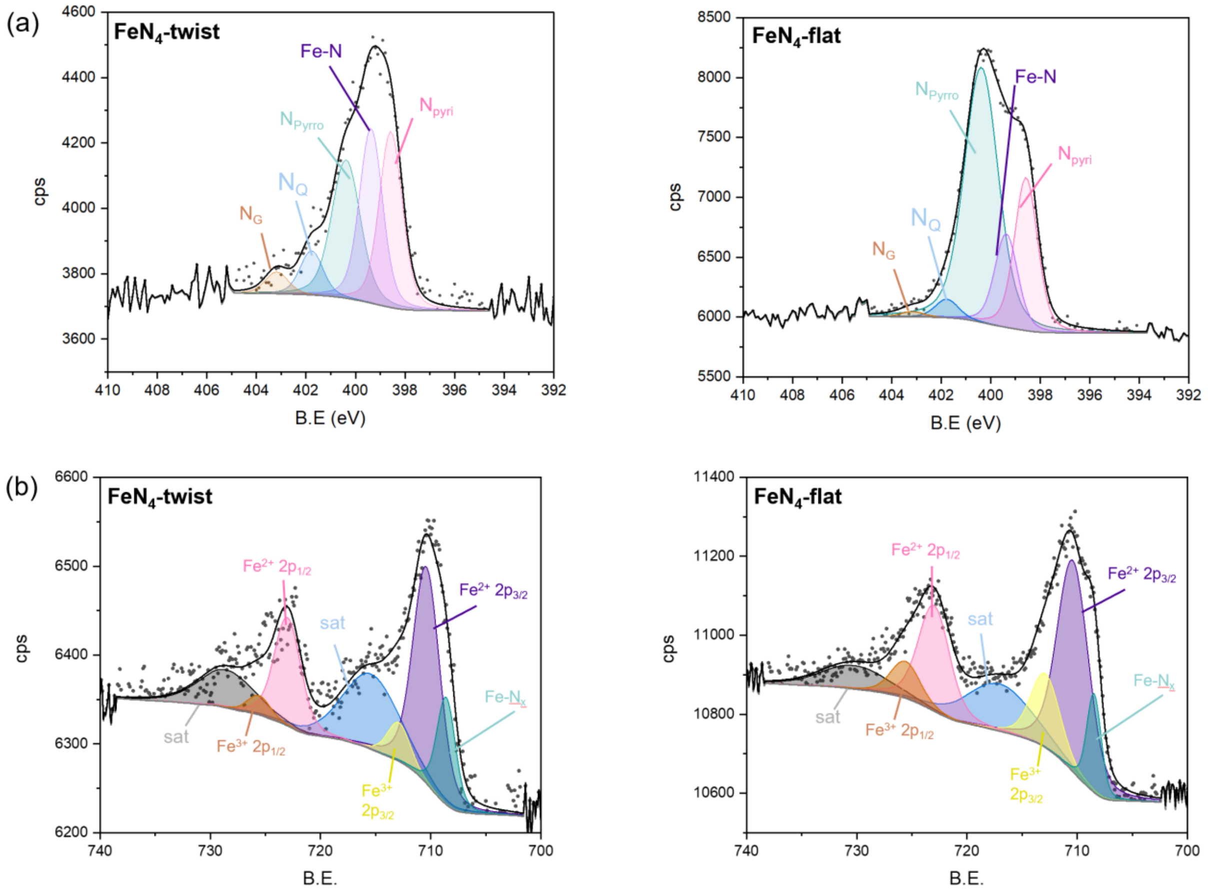

Figure S6: X-ray photoelectron spectroscopy (XPS) spectra in the (a) N 1s region, and (b) Fe 2p region. The N 1s spectra show a larger contribution from pyrrolic nitrogen in **FeN_4-flat** and a larger contribution for pyridinic nitrogen for **FeN_4-twist**. The Fe 2p spectra confirm the presence of Fe-N bonds, as well as a mixture of Fe^{2+} and Fe^{3+} oxidation states at the surface of the material.[62] This *ex situ* surface oxidation matches the estimate from the XAS edge position of 7120 eV (between +2.7 for a Fe_3O_4 reference and +3 for a Fe_2O_3 reference, as seen in Figure S17).

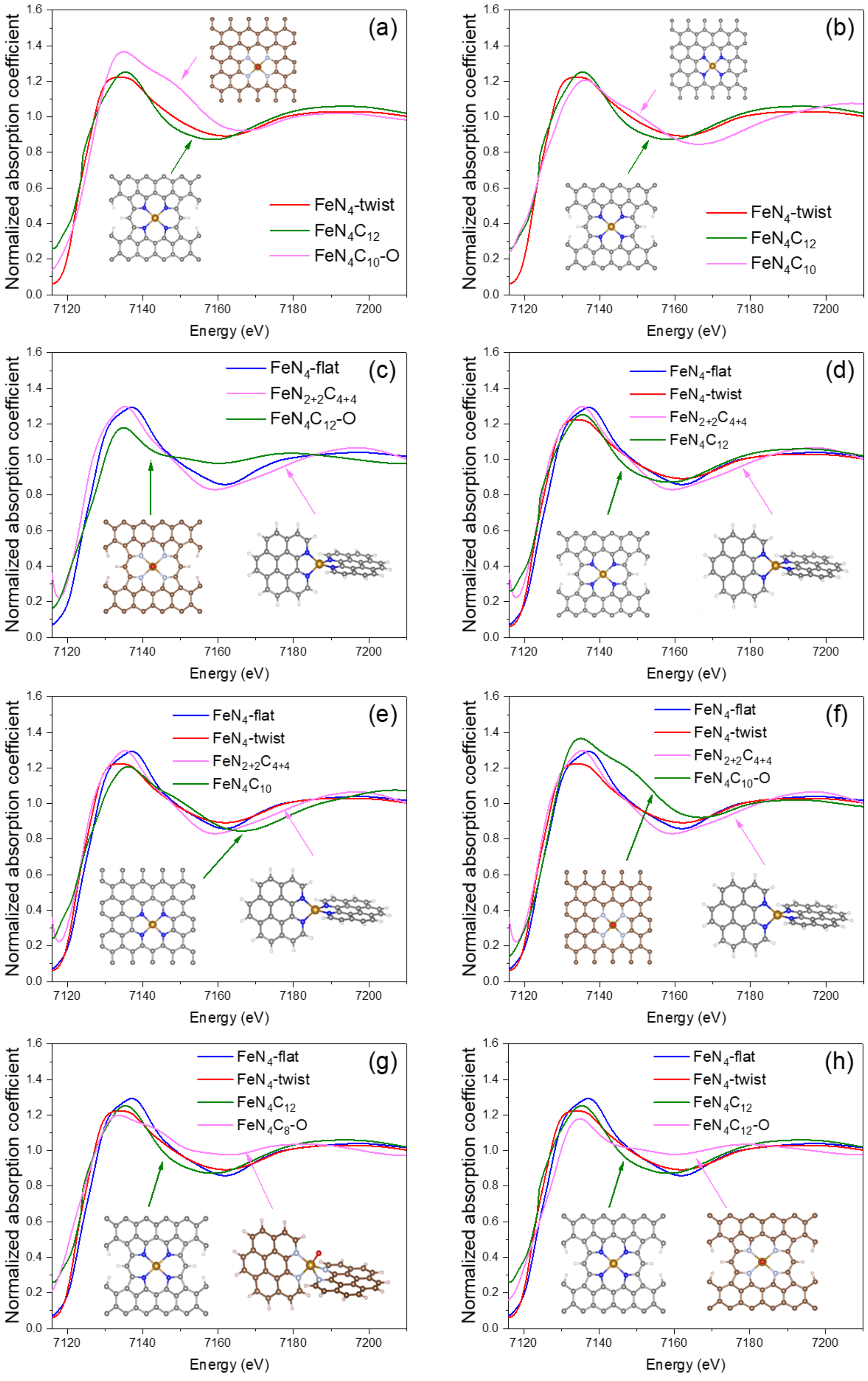

Figure S7: Other linear combination fits for **FeN_4-twist** and **FeN_4-flat**. In order to get a linear combination fit, one of the models should have lower E values than the experimental data and the other would have higher values. Only the models of FeN_4C_{12} and $FeN_{2+2}C_{4+4}$ had their spectrum below the experimental, therefore, only combinations considering one of these models were considered as possible fits.

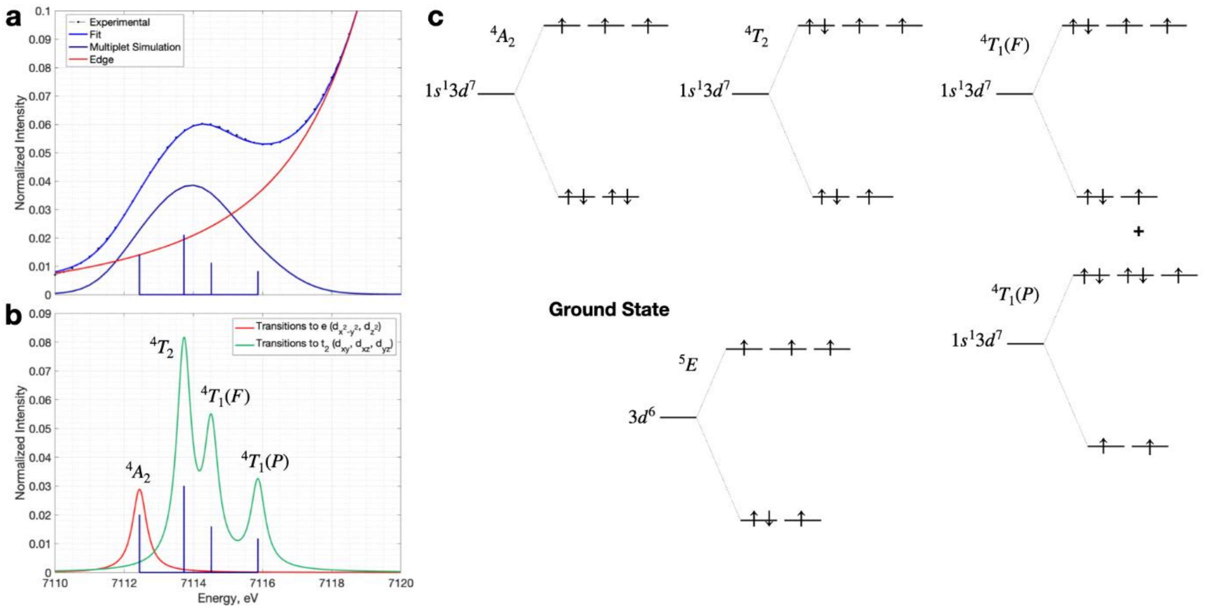

Figure S8: Representative crystal-field multiplet simulation fit for the pre-edge XANES of **FeN₄-twist** assuming a tetrahedral symmetry. Fitting values of 10Dq = –1.28 ± 0.07 with a scaling factor of 0.715 ± 0.035 for the Slater integrals from Hartree-Fock values (electron-electron repulsion), typical of tetrahedral high spin complexes with an intermediate level of covalency promoted by hybridization. (b) Identification of the states in the fit multiplet simulation, as described in (c). The fit simulation in (a) is projected to transitions to orbitals (*e* or t_2) starting from the identified ground state. The first transition is purely due to transitions to the *e* orbitals (red peak). The rest of the peaks are due to transitions into the t_2 orbitals, which are enhanced by 3d-4p hybridization. The projections in (b) are reproduced with less broadening to better reflect the orbital character of the transitions. Excitations to two $^4T_1$ multiplets are observed, the second one resulting from configuration interaction.

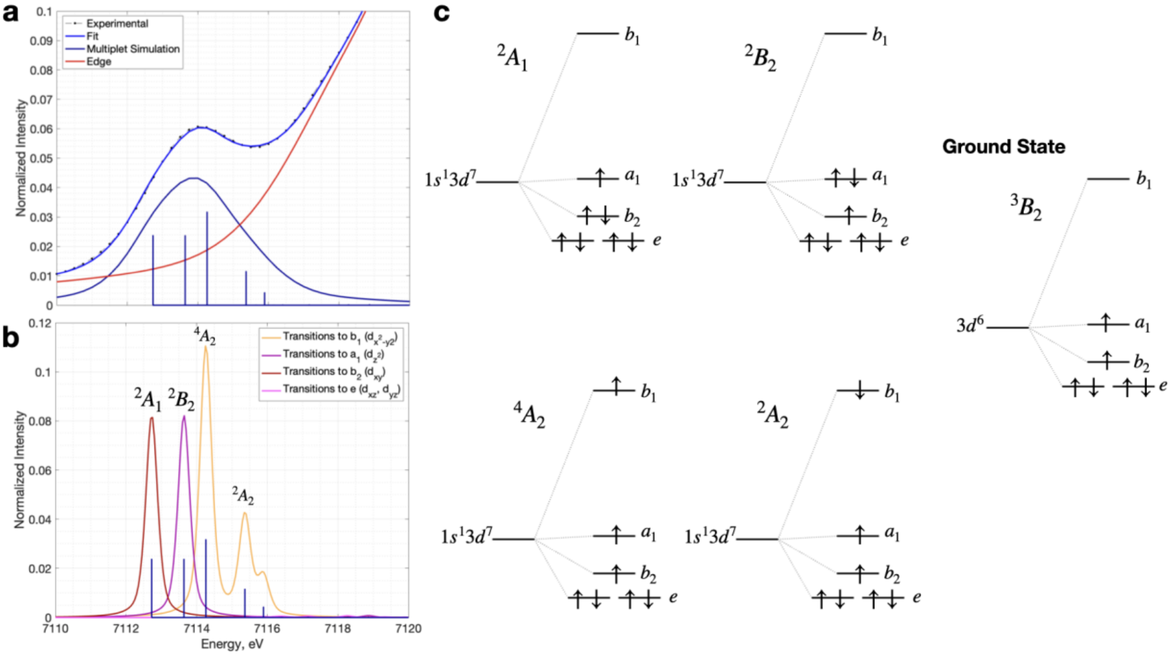

Figure S9: (a) Representative crystal field multiplet simulation fit for the pre-edge XANES of **FeN₄-flat** assuming D_{4h} symmetry. Crystal field fit parameters are 10Dq = 2.37 ± 0.26, D_σ = 0.57 ± 0.10 and D_τ = 0.14 ± 0.02 (similar to known parameters for Fe-phthalocyanine) are obtained with Slater integrals scaled to 46 ± 14% from Hartree-Fock values consistent to an intermediate spin complex. (b) Identification of multiplets in the final states, with details given in (c). Due to the large splitting between the a_1 (d_{z^2}) and b_1 ($d_{x^2-y^2}$) orbitals, it is more favorable to pair electrons in the a_1 (d_{z^2}) and b_2 (d_{xy}) orbitals and pay the price of electron-electron repulsion. Thus, the excitations to the b_1 orbital ($d_{x^2-y^2}$) are observed at higher energies. As this orbital was originally empty, the transition that gives the quartet ($^4A_2$) is energetically more favorable than the doublet because of the exchange with other electrons with the same spin in the 3*d* manifold.

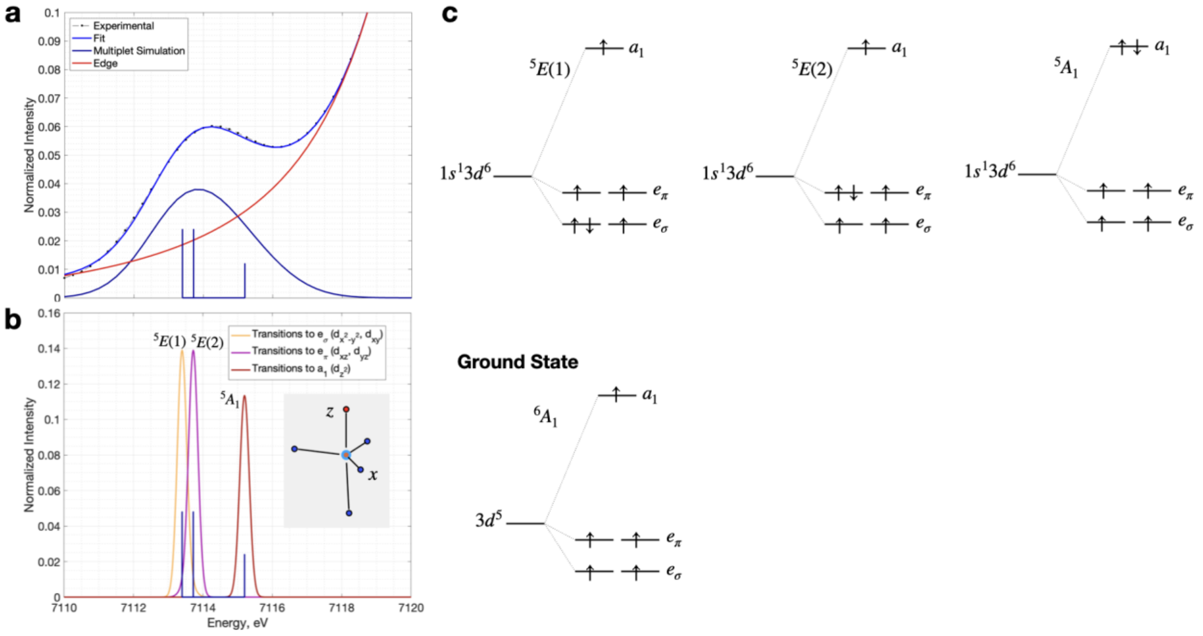

Figure S10: (a) Representative crystal field multiplet simulation fit for the pre-edge Fe XANES of $FeN_{2+2}C_{4+4}O$, the oxygenated site of **FeN_4-twist**. Obtained crystal field fit parameters are 10Dq = 0.31 ± 0.14, D_σ = –0.20 ± 0.07 and D_τ = –0.18 ± 0.03 with Slater integrals scaled to 64 ± 15% from Hartree-Fock values corresponding to a Fe(III) high spin complex with an approximate C_{3v} symmetry within a distorted bipyramidal trigonal environment (inset in b). (b) Identification of multiplets in the final state, with details given in (c). As the equatorial ligands are not lying completely in the *xy* plane, the value of 10Dq is slightly different than zero, which causes the two sets of *e* orbitals to be a linear combination of e_σ and e_π. Thus, the first two transitions are linear combinations to the $^5E(1)$ and $^5E(2)$ states.

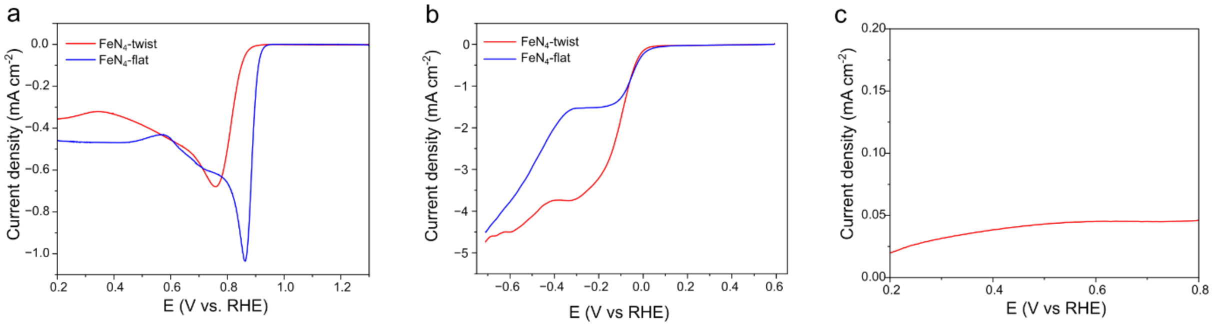

Figure S11: Can **FeN_4-twist** electrocatalyze other reactions? (a) Oxygen reduction LSV, in O_2-saturated 0.1 M KOH; **FeN_4-flat** is more efficient than **FeN_4-twist**. (b) Nitrite reduction LSV, in 10 mM $NaNO_2$, 0.15 M NaPBS, pH=6.7, **FeN_4-twist** shows higher current, but similar onset to **FeN_4-flat**. (c) Ammonia oxidation LSV on **FeN_4-twist**, in 2 M NH_3, 0.1 M KOH; no anodic current is observed in the range preceding to anodic carbon corrosion, showing that it cannot catalyze ammonia oxidation. All scans used 10 mV/s scan rate.

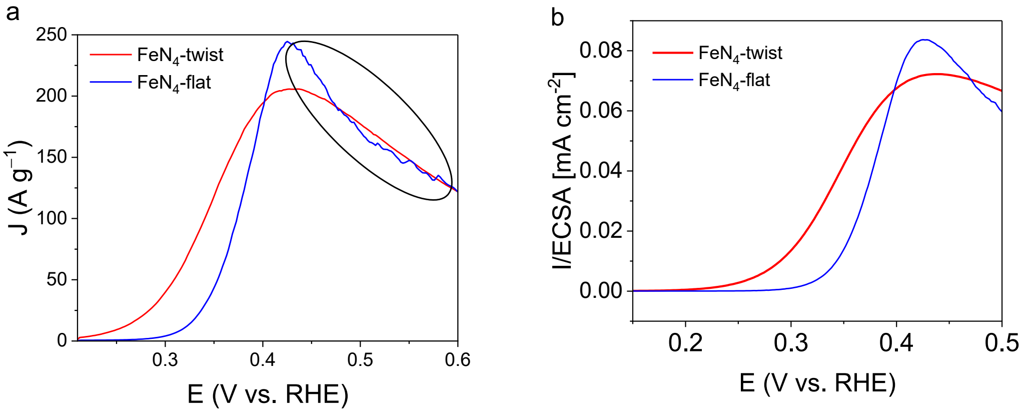

Figure S12: LSV curves of **FeN4-twist** and **FeN4-flat** normalized to (a) catalyst mass loading (b) their measured ECSA.

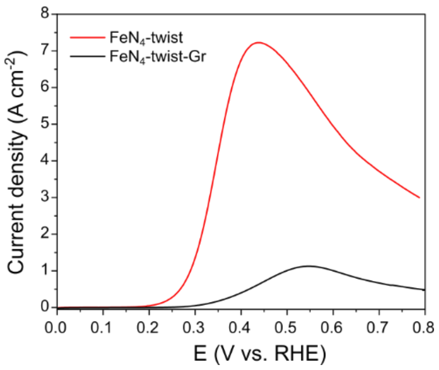

Figure S13: LSV of the **FeN_4-twist** and **FeN_4-twist-Gr** catalysts, where the respective iron precursors were deposited on either rGO (**FeN_4-twist**) or on graphite powder (**FeN_4-twist-Gr**). Recorded in in 1 M KOH and 20 mM hydrazine.

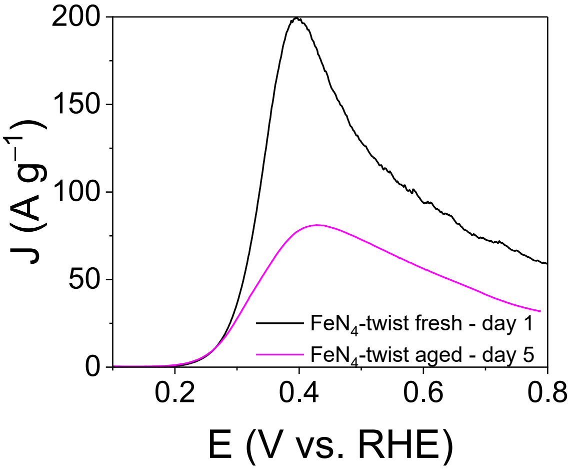

Figure S14: LSV of the HzOR electrocatalysis of **FeN_4-twist** catalyst (a) fresh after synthesis (day 1) and (b) after 5 days of storage in air, showing no change to the onset potential.

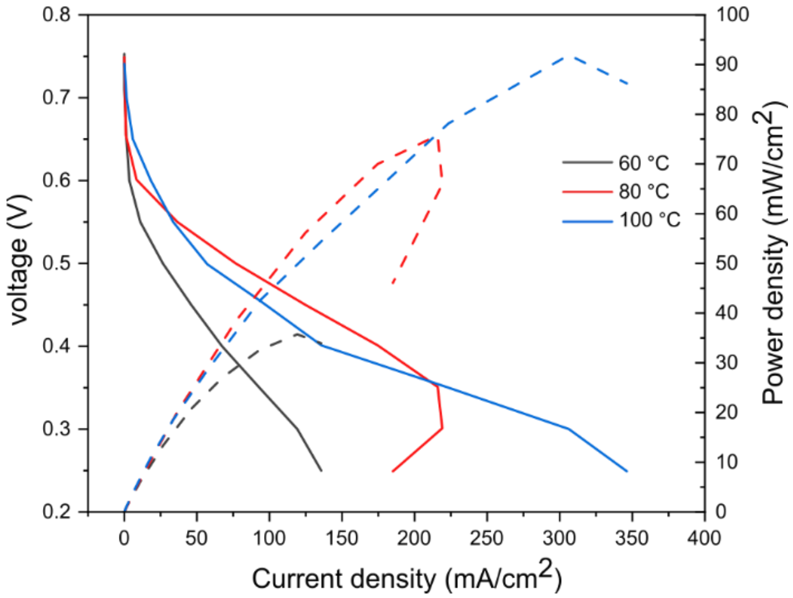

Figure S15: Direct hydrazine fuel cell employing a **FeN₄-flat** anode and a Pt/C cathode, loading 4 and 1 mg cm^{-2}, respectively. Inset: polarization curve in the kinetic region. Cell temperatures of 60, 80, and 100 °C with optimized cathode dew points; anode and cathode flow rates of 0.002 L min^{-1} and 0.5 L min^{-1}, respectively.

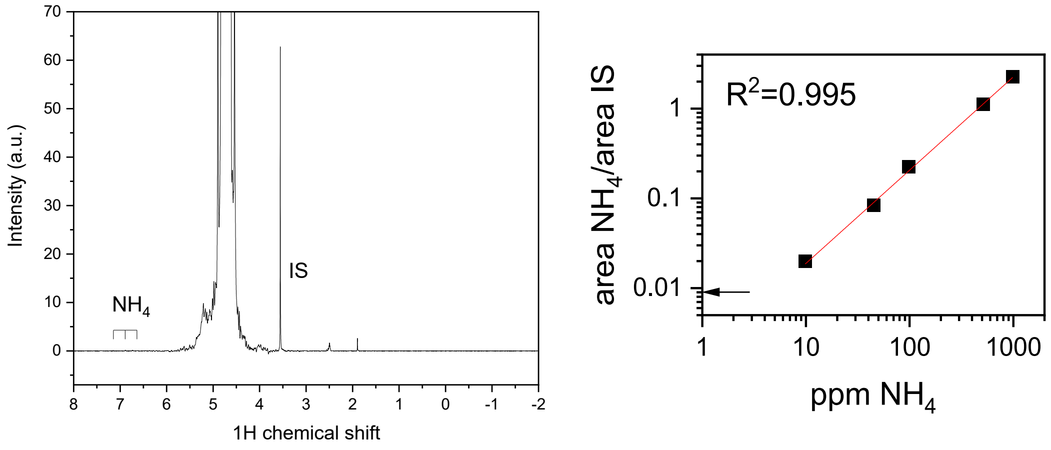

Figure S16: (a) NMR spectra of the exhaust solution of the **FeN4-twist** anode side of the fuel cell. (b) A calibration curve for ammonia. The value corresponding to the exhaust solution is marked with an arrow, where the ammonia content was below 10 ppm, the lower limit of the calibration curve.

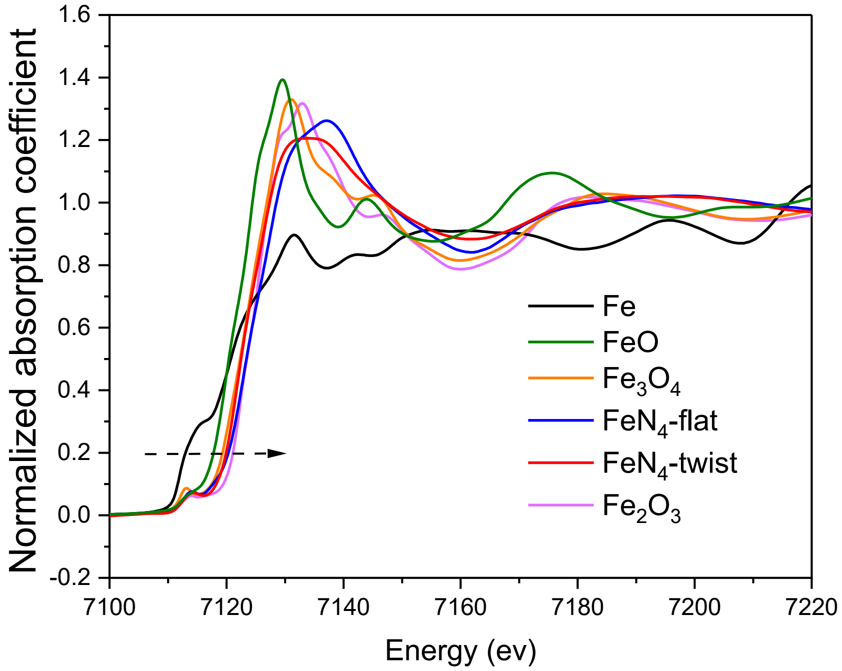

Figure S17: XANES spectra of the samples compared with different references, showing a monotonic progression of the edge versus the iron oxidation state, at the absorption coefficient marked by an arrow.

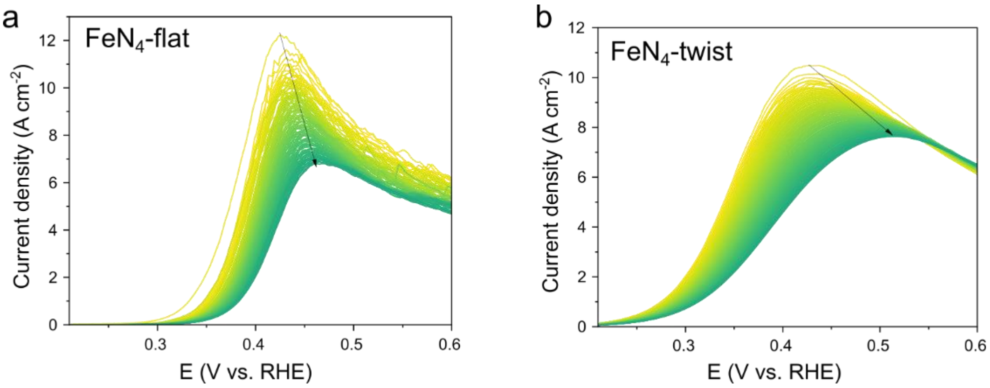

Figure S18*:* Stability of (a) **FeN_4-flat** and (b) **FeN_4-twist** over 100 cycles between 0.2–0.6 V vs. RHE at 10 mV/s. The onset potential of **FeN_4-flat** shifts by ~50 mV, while the onset of **FeN_4-twist** remains stable. The current density declines on both catalysts, but **FeN_4-twist** still surpasses **FeN_4-flat** after 100 cycles. Since the onset potential is unchanged and the current decline is accompanied by a slope change, it is possible that the deterioration of **FeN_4-twist** arises from increased resistance with bubble accumulation (due to enhanced bubbling). Meanwhile, for **FeN_4-flat**, the delay in onset potential could point to a different mechanism, involving degradation of the Fe-N_4 site.[63]

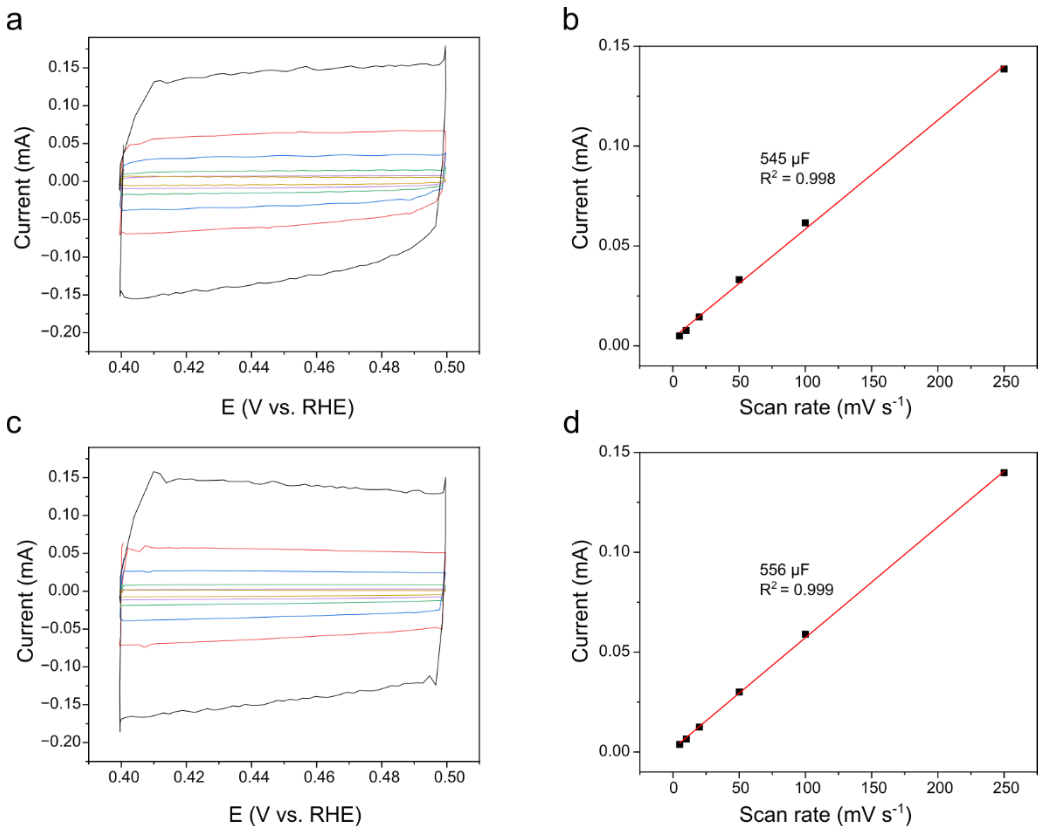

Figure S19: (a,c) CV scans at different scan rates in the non-faradaic region and (b,d) the linear regressions of current vs. scan rate, from the slope of which the capacitance is calculated. (a, b) **FeN_4-twist**; (c, d) **FeN_4-flat**.

References

1 T. Y. Burshtein, K. Tamakuwala, M. Sananis, I. Grinberg, N. R. Samala and D. Eisenberg, Understanding hydrazine oxidation electrocatalysis on undoped carbon, *Phys. Chem. Chem. Phys.*, 2022, **24**, 9897–9903.

2 K. Lagarec and D. G. Rancourt, 1998.

3 J. Lilloja, E. Kibena-Põldsepp, A. Sarapuu, J. C. Douglin, M. Käärik, J. Kozlova, P. Paiste, A. Kikas, J. Aruväli, J. Leis, V. Sammelselg, D. R. Dekel and K. Tammeveski, Transition-Metal- and Nitrogen-Doped Carbide-Derived Carbon/Carbon Nanotube Composites as Cathode Catalysts for Anion-Exchange Membrane Fuel Cells, *ACS Catal.*, 2021, **11**, 1920–1931.

4 A. S. Barbosa, A. L. G. Biancolli, A. J. C. Lanfredi, O. Rodrigues, F. C. Fonseca and E. I. Santiago, Enhancing the durability and performance of radiation-induced grafted low-density polyethylene-based anion-exchange membranes by controlling irradiation conditions, *Journal of Membrane Science*, 2022, **659**, 120804.

5 J. C. Douglin, R. K. Singh, A. C. Yang-Neyerlin, C. He, K. Yassin, H. A. Miller, M. V. Pagliaro, L. Capozzoli, E. Carbo-Argibay, S. Brandon, P. J. Ferreira, B. S. Pivovar and D. R. Dekel, Elucidating the degradation mechanisms of Pt-free anode anion-exchange membrane fuel cells after durability testing, *Journal of Materials Chemistry A*, 2024, **12**, 10435–10448.

6 M. Faour, K. Yassin and D. R. Dekel, Anion-Exchange Membrane Oxygen Separator, *ACS Org. Inorg. Au*, 2024, **4**, 498–503.

7 J. Xue, J. C. Douglin, K. Yassin, T. Huang, H. Jiang, J. Zhang, Y. Yin, D. R. Dekel and M. D. Guiver, High-temperature anion-exchange membrane fuel cells with balanced water management and enhanced stability, *Joule*, 2024, **8**, 1457–1477.

8 J. C. Douglin, A. Sekar, R. K. Singh, Z. Chen, J. Li and D. R. Dekel, Hydrogenated TiO2 Carbon Support for PtRu Anode Catalyst in High-Performance Anion-Exchange Membrane Fuel Cells, *Small*, 2024, **20**, 2307497.

9 J. Xue, J. C. Douglin, T. Huang, H. Jiang, J. Zhang, Y. Yin, D. R. Dekel and M. D. Guiver, Chain entanglement and free-volume effects in branched poly(arylene piperidinium) anion-exchange membranes: Random-branched versus end-branched, *Journal of Membrane Science*, 2025, **717**, 123519.

10 K. Yassin, J. C. Douglin, I. G. Rasin, P. G. Santori, B. Eriksson, N. Bibent, F. Jaouen, S. Brandon and D. R. Dekel, The effect of membrane thickness on AEMFC Performance: An integrated theoretical and experimental study, *Energy Conversion and Management*, 2022, **270**, 116203.

11 J. C. Douglin, K. Vijaya Sankar, A. L. G. Biancolli, E. I. Santiago, Y. Tsur and D. R. Dekel, Quantifying the Resistive Losses of the Catalytic Layers in Anion-Exchange Membrane Fuel Cells, *ChemSusChem*, 2023, **16**, e202301080.

12 J. C. Douglin, J. A. Zamora Zeledón, M. E. Kreider, R. K. Singh, M. B. Stevens, T. F. Jaramillo and D. R. Dekel, High-performance ionomerless cathode anion-exchange membrane fuel cells with ultra-low-loading Ag–Pd alloy electrocatalysts, *Nat Energy*, 2023, **8**, 1262–1272.

13 J. C. Douglin, R. K. Singh, S. Haj-Bsoul, S. Li, J. Biemolt, N. Yan, J. R. Varcoe, G. Rothenberg and D. R. Dekel, A high-temperature anion-exchange membrane fuel cell with a critical raw material-free cathode, *Chemical Engineering Journal Advances*, 2021, **8**, 100153.

14 M. V. Pagliaro, C. Wen, B. Sa, B. Liu, M. Bellini, F. Bartoli, S. Sahoo, R. K. Singh, S. P. Alpay, H. A. Miller and D. R. Dekel, Improving Alkaline Hydrogen Oxidation Activity of Palladium through Interactions with Transition-Metal Oxides, *ACS Catal.*, 2022, **12**, 10894–10904.

15 D. Menga, F. E. Wagner and T.-P. Fellinger, Life cycle of single atom catalysts: a Mössbauer study on degradation and reactivation of tetrapyrrolic Fe–N–C powders, *Materials Horizons*, 2023, **10**, 5577–5583.

16 A. Braun, L. B. Gee, M. W. Mara, E. A. Hill, T. Kroll, D. Nordlund, D. Sokaras, P. Glatzel, B. Hedman, K. O. Hodgson, A. S. Borovik, M. L. Baker and E. I. Solomon, X-ray Spectroscopic Study of the Electronic Structure of a Trigonal High-Spin Fe(IV)═O Complex Modeling Non-Heme Enzyme Intermediates and Their Reactivity, *J. Am. Chem. Soc.*, 2023, **145**, 18977–18991.

17 M. W. Haverkort, M. Zwierzycki and O. K. Andersen, Multiplet ligand-field theory using Wannier orbitals, *Phys. Rev. B*, 2012, **85**, 165113.

18 V. Vercamer, M. O. J. Y. Hunault, G. Lelong, M. W. Haverkort, G. Calas, Y. Arai, H. Hijiya, L. Paulatto, C. Brouder, M.-A. Arrio and A. Juhin, Calculation of optical and K pre-edge absorption spectra for ferrous iron of distorted sites in oxide crystals, *Phys. Rev. B*, 2016, **94**, 245115.

19 T. Kroll, M. L. Baker, S. A. Wilson, M. Lundberg, A. Juhin, M.-A. Arrio, J. J. Yan, L. B. Gee, A. Braun, T.-C. Weng, D. Sokaras, B. Hedman, K. O. Hodgson and E. I. Solomon, Effect of 3d/4p Mixing on 1s2p Resonant Inelastic X-ray Scattering: Electronic Structure of Oxo-Bridged Iron Dimers, *J. Am. Chem. Soc.*, 2021, **143**, 4569–4584.

20 Y.-C. Wang, L.-Y. Wan, P.-X. Cui, L. Tong, Y.-Q. Ke, T. Sheng, M. Zhang, S.-H. Sun, H.-W. Liang, Y.-S. Wang, K. Zaghib, H. Wang, Z.-Y. Zhou and J. Yuan, Porous Carbon Membrane-Supported Atomically Dispersed Pyrrole-Type Fe�N4 as Active Sites for Electrochemical Hydrazine Oxidation Reaction, *Small*, 2020, **16**, 2002203.

21 J. Zhang, Y. Wang, C. Yang, S. Chen, Z. Li, Y. Cheng, H. Wang, Y. Xiang, S. Lu and S. Wang, Elucidating the electro-catalytic oxidation of hydrazine over carbon nanotube-based transition metal single atom catalysts, *Nano Res.*, 2021, **14**, 4650–4657.

22 Q. Yu, X. Liu, G. Liu, X. Wang, Z. Li, B. Li, Z. Wu and L. Wang, Constructing Three-Phase Heterojunction with 1D/3D Hierarchical Structure as Efficient Trifunctional Electrocatalyst in Alkaline Seawater, *Advanced Functional Materials*, 2022, **32**, 2205767.

23 K. Asazawa, T. Sakamoto, S. Yamaguchi, K. Yamada, H. Fujikawa, H. Tanaka and K. Oguro, Study of Anode Catalysts and Fuel Concentration on Direct Hydrazine Alkaline Anion-Exchange Membrane Fuel Cells, *J. Electrochem. Soc.*, 2009, **156**, B509.

24 S. Bae, J. Park, Y. Hwang, J.-S. Park, J. Lee and B. Jeong, Steam activation of Fe-N-C catalyst for advanced power performance of alkaline hydrazine fuel cells, *Journal of Energy Chemistry*, 2022, **64**, 276–285.

25 S. Bae, J. Park, S. Bong, J.-S. Park, B. Jeong and J. Lee, Pore surface engineering of FeNC for outstanding power density of alkaline hydrazine fuel cells, *Chemical Engineering Journal*, 2023, 147522.

26 T. Sakamoto, K. Asazawa, J. Sanabria-Chinchilla, U. Martinez, B. Halevi, P. Atanassov, P. Strasser and H. Tanaka, Combinatorial discovery of Ni-based binary and ternary catalysts for hydrazine electrooxidation for use in anion exchange membrane fuel cells, *Journal of Power Sources*, 2014, **247**, 605–611.

27 A. Serov, M. Padilla, A. J. Roy, P. Atanassov, T. Sakamoto, K. Asazawa and H. Tanaka, Anode Catalysts for Direct Hydrazine Fuel Cells: From Laboratory Test to an Electric Vehicle, *Angewandte Chemie*, 2014, **126**, 10504–10507.

28 B. Filanovsky, E. Granot, I. Presman, I. Kuras and F. Patolsky, Long-term room-temperature hydrazine/air fuel cells based on low-cost *nanotextured* Cu–Ni catalysts, *Journal of Power Sources*, 2014, **246**, 423–429.

29 E. Granot, B. Filanovsky, I. Presman, I. Kuras and F. Patolsky, Hydrazine/air direct-liquid fuel cell based on nanostructured copper anodes, *Journal of Power Sources*, 2012, **204**, 116–121.

30 Z. Lu, M. Sun, T. Xu, Y. Li, W. Xu, Z. Chang, Y. Ding, X. Sun and L. Jiang, Superaerophobic Electrodes for Direct Hydrazine Fuel Cells, *Advanced Materials*, DOI:10.1002/adma.201500064.

31 T. Sakamoto, K. Asazawa, U. Martinez, B. Halevi, T. Suzuki, S. Arai, D. Matsumura, Y. Nishihata, P. Atanassov and H. Tanaka, Electrooxidation of hydrazine hydrate using Ni–La catalyst for anion exchange membrane fuel cells, *Journal of Power Sources*, 2013, **234**, 252–259.

32 X. Liu, J. He, S. Zhao, Y. Liu, Z. Zhao, J. Luo, G. Hu, X. Sun and Y. Ding, Self-powered H2 production with bifunctional hydrazine as sole consumable, *Nat Commun*, 2018, **9**, 4365.

33 J. Park, S. Bae, J.-S. Park, S. Bong and J. Lee, Crusty-Structured Cu@NiCo Nanoparticles as Anode Catalysts in Alkaline Fuel Cells, *ACS Appl. Nano Mater.*, 2021, **4**, 8145–8153.

34 Q. Qian, J. Zhang, J. Li, Y. Li, X. Jin, Y. Zhu, Y. Liu, Z. Li, A. El-Harairy, C. Xiao, G. Zhang and Y. Xie, Artificial Heterointerfaces Achieve Delicate Reaction Kinetics towards Hydrogen Evolution and Hydrazine Oxidation Catalysis, *Angewandte Chemie*, 2021, **133**, 6049–6058.
35 H. Sun, L. Gao, A. Kumar, Z. Cao, Z. Chang, W. Liu and X. Sun, Superaerophobic CoP Nanowire Arrays as a Highly Effective Anode Electrocatalyst for Direct Hydrazine Fuel Cells, *ACS Appl. Energy Mater.*, 2022, **5**, 9455–9462.
36 M. Zhang, J. Zhu, B. Liu, Y. Hou, C. Zhang, J. Wang and J. Niu, Ultrafine Co 6 W 6 C as an efficient anode catalyst for direct hydrazine fuel cells, *Chemical Communications*, 2021, **57**, 10415–10418.
37 J. Deng, X. Li, S. Imhanria, K. Chen, X. Deng and W. Wang, Molybdenum carbide-nitrogen doped carbon composites as effective non-precious electrocatalyst for direct hydrazine fuel cell, *Electrochimica Acta*, 2021, **384**, 138417.
38 R. Crisafulli, D. F. de Paula, S. C. Zignani, L. Spadaro, A. Palella, S. Boninelli, J. A. Dias and J. J. Linares, Promoting Effect of Cu on Pd Applied to the Hydrazine Electro-Oxidation and Direct Hydrazine Fuel Cells, *Catalysts*, 2022, **12**, 1639.
39 Y. Wang, Q. Wang, L. Wan, Y. Han, Y. Hong, L. Huang, X. Yang, Y. Wang, K. Zaghib and Z. Zhou, KOH-doped polybenzimidazole membrane for direct hydrazine fuel cell, *Journal of Colloid and Interface Science*, 2020, **563**, 27–32.
40 Y. Li, J. Zhang, Y. Liu, Q. Qian, Z. Li, Y. Zhu and G. Zhang, Partially exposed RuP2 surface in hybrid structure endows its bifunctionality for hydrazine oxidation and hydrogen evolution catalysis, *Science Advances*, 2020, **6**, eabb4197.
41 K. Pang, Y. Tang, C. Qiu, M. Zhang, A. Tayal, S. Feng, C. Long, Y. Wang, J. Chang, B. Pang, A. Sikdar, S. S. Garakani, Y. Zhang, H. Wang, W. Zhang, G. Luo, Y. Wang and J. Yuan, Redirecting configuration of atomically dispersed selenium catalytic sites for efficient hydrazine oxidation, *Matter*, 2024, **7**, 655–667.
42 J. Gao, W. Yu, J. Liu, L. Qin, H. Cheng, X. Cui and L. Jiang, Regulation of hydrogen binding energy via oxygen vacancy enables an efficient trifunctional Rh-Rh2O3 electrocatalyst for fuel cells and water splitting, *Journal of Colloid and Interface Science*, 2024, **664**, 766–778.
43 K. Yamada, K. Yasuda, H. Tanaka, Y. Miyazaki and T. Kobayashi, Effect of anode electrocatalyst for direct hydrazine fuel cell using proton exchange membrane, *Journal of Power Sources*, 2003, **122**, 132–137.
44 On the promotional effect of Cu on Pt for hydrazine electrooxidation in alkaline medium, *Applied Catalysis B: Environmental*, 2018, **236**, 36–44.
45 F. Zhao, S. Nie, L. Wu, Q. Yuan and X. Wang, Porous, Ultrathin PtAgBiTe Nanosheets for Direct Hydrazine Hydrate Fuel Cell Devices, *Advanced Materials*, 2023, **35**, 2303672.
46 T. Sakamoto, A. Serov, T. Masuda, M. Kamakura, K. Yoshimoto, T. Omata, H. Kishi, S. Yamaguchi, A. Hori, Y. Horiuchi, T. Terada, K. Artyushkova, P. Atanassov and H. Tanaka, Highly durable direct hydrazine hydrate anion exchange membrane fuel cell, *Journal of Power Sources*, 2018, **375**, 291–299.
47 Magic hybrid structure as multifunctional electrocatalyst surpassing benchmark Pt/C enables practical hydrazine fuel cell integrated with energy-saving H2 production, *eScience*, 2022, **2**, 416–427.
48 G. L. Soloveichik, Liquid fuel cells, *Beilstein J. Nanotechnol.*, 2014, **5**, 1399–1418.
49 D. Cao and S. H. Bergens, A direct 2-propanol polymer electrolyte fuel cell, *Journal of Power Sources*, 2003, **124**, 12–17.
50 J. Wang, S. Wasmus and R. F. Savinell, Evaluation of Ethanol, 1-Propanol, and 2-Propanol in a Direct Oxidation Polymer-Electrolyte Fuel Cell: A Real-Time Mass Spectrometry Study, *J. Electrochem. Soc.*, 1995, **142**, 4218.
51 Z. Qi and A. Kaufman, Liquid-feed direct oxidation fuel cells using neat 2-propanol as fuel, *Journal of Power Sources*, 2003, **118**, 54–60.

52 M. Brodt, K. Müller, J. Kerres, I. Katsounaros, K. Mayrhofer, P. Preuster, P. Wasserscheid and S. Thiele, The 2-Propanol Fuel Cell: A Review from the Perspective of a Hydrogen Energy Economy, *Energy Technology*, 2021, **9**, 2100164.
53 R. G. C. S. Reis, C. A. Martins and G. A. Camara, The Electrooxidation of 2-Propanol: An Example of an Alternative Way to Look at In Situ FTIR Data, *Electrocatal*, 2010, **1**, 116–121.
54 T. Y. Burshtein, E. M. Farber, K. Ojha and D. Eisenberg, Revealing structure–activity links in hydrazine oxidation: doping and nanostructure in carbide–carbon electrocatalysts, *Journal of Materials Chemistry A*, 2019, **7**, 23854–23861.
55 T. Y. Burshtein, D. Aias, J. Wang, M. Sananis, E. M. Farber, O. M. Gazit, I. Grinberg and D. Eisenberg, Fe–N–C electrocatalysts in the oxygen and nitrogen cycles in alkaline media: the role of iron carbide, *Physical Chemistry Chemical Physics*, 2021, **23**, 26674–26679.
56 Y. Zheng, F. He, M. Chen, J. Zhang, G. Hu, D. Ma, J. Guo, H. Fan, W. Li and X. Hu, Mimicking Hydrazine Dehydrogenase for Efficient Electrocatalytic Oxidation of N2H4 by Fe–NC, *ACS Appl. Mater. Interfaces*, 2020, **12**, 38183–38191.
57 K. Ojha, E. M. Farber, T. Y. Burshtein and D. Eisenberg, A Multi-Doped Electrocatalyst for Efficient Hydrazine Oxidation, *Angewandte Chemie International Edition*, 2018, **57**, 17168–17172.
58 I. Salton, K. Ioffe, T. Y. Burshtein, E. M. Farber, N. M. Seraphim, N. Segal and D. Eisenberg, Lanthanoid coordination compounds as diverse self-templating agents towards hierarchically porous Fe–N–C electrocatalysts, *Materials Advances*, 2022, **3**, 7937–7945.
59 Y. Wang, Z. Chen, H. Wu, F. Xiao, E. Cao, S. Du, Y. Wu and Z. Ren, Self-Assembly-Induced Mosslike Fe2O3 and FeP on Electro-oxidized Carbon Paper for Low-Voltage-Driven Hydrogen Production Plus Hydrazine Degradation, *ACS Sustainable Chem. Eng.*, 2018, **6**, 15727–15736.
60 L. Ni, C. Gallenkamp, S. Paul, M. Kübler, P. Theis, S. Chabbra, K. Hofmann, E. Bill, A. Schnegg, B. Albert, V. Krewald and U. I. Kramm, Active Site Identification in FeNC Catalysts and Their Assignment to the Oxygen Reduction Reaction Pathway by In Situ 57Fe Mössbauer Spectroscopy, *Advanced Energy and Sustainability Research*, 2021, **2**, 2000064.
61 Ll. Casas, A. Roig, E. Molins, J. M. Grenèche, J. Asenjo and J. Tejada, Iron oxide nanoparticles hosted in silica aerogels, *Appl Phys A*, 2002, **74**, 591–597.
62 K. Artyushkova, A. Serov, H. Doan, N. Danilovic, C. B. Capuano, T. Sakamoto, H. Kishi, S. Yamaguchi, S. Mukerjee and P. Atanassov, Application of X-ray photoelectron spectroscopy to studies of electrodes in fuel cells and electrolyzers, *Journal of Electron Spectroscopy and Related Phenomena*, DOI:10.1016/j.elspec.2017.12.006.
63 J. Li, M. T. Sougrati, A. Zitolo, J. M. Ablett, I. C. Oğuz, T. Mineva, I. Matanovic, P. Atanassov, Y. Huang, I. Zenyuk, A. Di Cicco, K. Kumar, L. Dubau, F. Maillard, G. Dražić and F. Jaouen, Identification of durable and non-durable FeNx sites in Fe–N–C materials for proton exchange membrane fuel cells, *Nat Catal*, 2021, **4**, 10–19.
